# Single-molecule detection of dihydroazulene photo-thermal reaction using break junction technique

**DOI:** 10.1038/ncomms15436

**Published:** 2017-05-22

**Authors:** Cancan Huang, Martyn Jevric, Anders Borges, Stine T. Olsen, Joseph M. Hamill, Jue-Ting Zheng, Yang Yang, Alexander Rudnev, Masoud Baghernejad, Peter Broekmann, Anne Ugleholdt Petersen, Thomas Wandlowski, Kurt V. Mikkelsen, Gemma C. Solomon, Mogens Brøndsted Nielsen, Wenjing Hong

**Affiliations:** 1 State Key Laboratory of Physical Chemistry of Solid States, College of Chemistry and Chemical Engineering, Collaborative Innovation Center of Chemistry for Energy Materials, Xiamen University, 361005 Xiamen, China; 2 Department of Chemistry and Biochemistry, University of Bern, Freiestrasse 3, CH-3012 Bern, Switzerland; 3 Department of Chemistry and Nano-Science Center, University of Copenhagen, Universitetsparken 5, DK-2100 Copenhagen Ø, Denmark

## Abstract

Charge transport by tunnelling is one of the most ubiquitous elementary processes in nature. Small structural changes in a molecular junction can lead to significant difference in the single-molecule electronic properties, offering a tremendous opportunity to examine a reaction on the single-molecule scale by monitoring the conductance changes. Here, we explore the potential of the single-molecule break junction technique in the detection of photo-thermal reaction processes of a photochromic dihydroazulene/vinylheptafulvene system. Statistical analysis of the break junction experiments provides a quantitative approach for probing the reaction kinetics and reversibility, including the occurrence of isomerization during the reaction. The product ratios observed when switching the system in the junction does not follow those observed in solution studies (both experiment and theory), suggesting that the junction environment was perturbing the process significantly. This study opens the possibility of using nano-structured environments like molecular junctions to tailor product ratios in chemical reactions.

## Abstract (web summary)

The conductance across single-molecule junctions is highly dependent on the electronic properties of the molecule in question. Here the authors use this fact to monitor a photo-thermal reaction by analysing break junction data, and observe significant differences compared to solution state behaviour.

The switching of photochromic molecules is accompanied by changes in electronic, structural and/or chemical properties, making photoswitches versatile building blocks for potential applications in materials science, electronics and biotechnology^1^,^2^,^3^. Progress in this area requires detailed understanding of these molecules and various analytical techniques have been used to analyse the photoreaction process, such as ultraviolet–visible (UV–Vis) spectroscopy, fluorescence spectroscopy^4^,^5^ and Raman spectroscopy^6^,^7^. As a potential analytical tool, the single-molecule break junction technique has been employed to study the conductance–structure correlation of a large range of molecular components in the past years^8^,^9^,^10^,^11^,^12^,^13^,^14^. More importantly, the break-junction techniques could also provide a means to statistically quantify the photo-reaction kinetics of a single-molecule device connected between two electrodes, which may offer some new understanding beyond measuring an ensemble of molecules as in other spectroscopies.

We became interested in exploring the dihydroazulene (**dha**)/vinylheptafulvene (**vhf**) system^15^,^16^ as previous studies have shown light-controlled conductance switching of **dha** derivatives in a silver nanogap fabricated using sublimable molecules and also bias-induced switching for a junction operating in the Coulomb blockade regime^17^,^18^. Fabrication of junctions by sublimation puts some constraints on the molecules that can be studied in regard to molecular weight and stability. The break-junction technique employs a solution of the molecules and it has allowed us to achieve charge transport properties of larger **dha** molecules by incorporating anchoring groups (−SAc) at each end of the molecule to control the anchoring configuration.

We employ the mechanically controllable break junction (MCBJ) technique to detect the photo-thermal reaction processes of a tailor-made photochromic **dha**/**vhf** system. Three distinguishable conductance states are observed experimentally, while reversible and distinct changing of the single molecular conductance is observed between two of the states. Benefiting from the well-distinguished conductance states, the reaction kinetics and reversibility could be studied via statistical analysis of the conductance–distance traces, and it is found that the reaction in the junction does not follow those observed in solution, which agrees well with the energy calculation. Density functional transport calculations reproduce the relative conductance of these molecular states and identify a changing destructive interference effect as being responsible for the large span in conductance.

## Results

### Single-molecule conductance measurement

**Figure 1:**
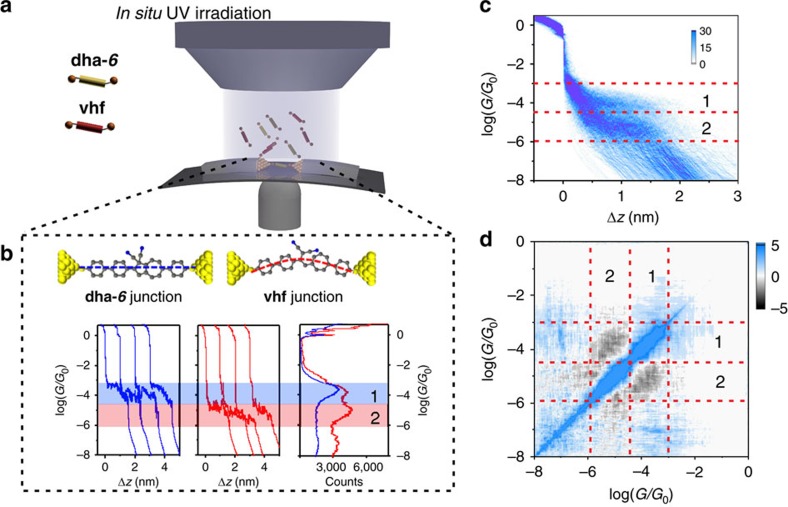
Single-molecule conductance measurements. (**a**) Schematic of the mechanically controllable break junction (MCBJ) measurements during the photo-conversion of dihydroazulene under the *in situ* ultraviolet (UV) irradiation. The solution contains dihydroazulene (**dha-*****6***) and vinylheptafulvene (**vhf**). (**b**) Typical individual conductance–distance traces recorded in break junction measurements; blue for the **dha-*****6*** junction and red for the **vhf** junction. The applied bias potential is 100 mV. (**c**) 2D conductance–displacement histogram constructed from 1,000 conductance–distance traces, the conductance region 10^−3.0^∼10^−4.5^ *G*_0_ is defined as region 1, and the conductance region 10^−4.5^∼10^−6.0^ *G*_0_ is defined as region 2. Linear scaling is applied for the colour bar. (**d**) 2D covariance histogram constructed from 1,000 conductance–distance traces.

We measured the single-molecule conductance of the target molecules using the MCBJ technique under ambient conditions^19^,^20^. The MCBJ measurements proceeded as follows: the contacted point of the two electrodes was repeatedly broken and reformed in the solution containing 0.2 mM target molecule under the control of piezo-stack movement. During the breaking process, the anchoring groups (thioacetates) can contact the surface of gold electrodes via Au–S bond and bridge the molecule between the separated electrodes to form the Au-molecule-Au junction. Under a fixed bias (100 mV), the current through the junction was recorded and is used for the further analysis. Figure 1b shows the typical conductance traces plotted on a logarithmic scale. The plateaus with a conductance value below *G*_0_ (conductance quantum) are assigned to molecular junctions. Hundreds to thousands of conductance traces are used to construct the one-dimensional (1D) histograms and two-dimensional (2D) histograms.

To probe the photo-conversion process of **dha-*****6***, we introduced the *in situ* UV irradiation system to the MCBJ setup. In the initial state (**dha-*****6*** solution without UV irradiation), all the molecular junctions are formed by trapping the **dha-*****6*** into the nanogaps (**dha-*****6*** junction). The conductance traces show the plateau in the range 10^−3.0^∼10^−4.5^ *G*_0_, and the maximum conductance peak around 10^−3.7±0.1^ *G*_0_ is obtained by a Gaussian fitting of the constructed 1D histogram. With UV irradiation (365 nm), the **dha-*****6*** undergoes a ring-opening process of the five-membered ring to form **vhf**. Thus, both **dha-*****6*** and **vhf** exist in the solution simultaneously. During the conductance measurements, two kinds of conductance traces are observed, one with a plateau around 10^−3.0^∼10^−4.5^ *G*_0_ (marked as region 1) assigned to **dha-*****6*** junction (blue traces) and the other with a plateau around 10^−4.5^∼10^−6^ *G*_0_ (marked as region 2), which is ascribed to **vhf** junctions. The 1D conductance histogram is plotted in Fig. 1b (right part), showing two distinct conductance peaks. The constructed 2D conductance–displacement histogram (Fig. 1c) also shows two well-distinguished conductance features with more than one order of magnitude (1.4) conductance difference between them and almost the same plateau length. Moreover, we analysed the correlation between these two kinds of conductance traces by compiling the 2D covariance histogram in Fig. 1d (ref. 21). The significant anti-correlation in the intersection of region 1 and region 2 indicates that the molecular junction is constructed either by **dha-*****6*** or **vhf** for each stretching cycle. Therefore, we can extract each type of conductance trace to quantitatively determine the percentages of **dha-*****6*** and **vhf** in solution.

### Photo-thermal reaction kinetics

To further investigate the photoreaction process of the **dha-*****6***, we studied the reaction kinetics with *in situ* UV irradiation by statistical analysis of the percentage of **dha-*****6*** as a function of time. Based on the anti-correlation between **dha-*****6*** junction and **vhf** junction mentioned above, the ratio of **dha-*****6*****/vhf** or the content of each component (**dha-*****6*** **or vhf**) could be determined from counting the traces presenting each conductance character, although there are around 5–10% traces exhibiting direct tunnelling without forming a molecular junction.

**Figure 2:**
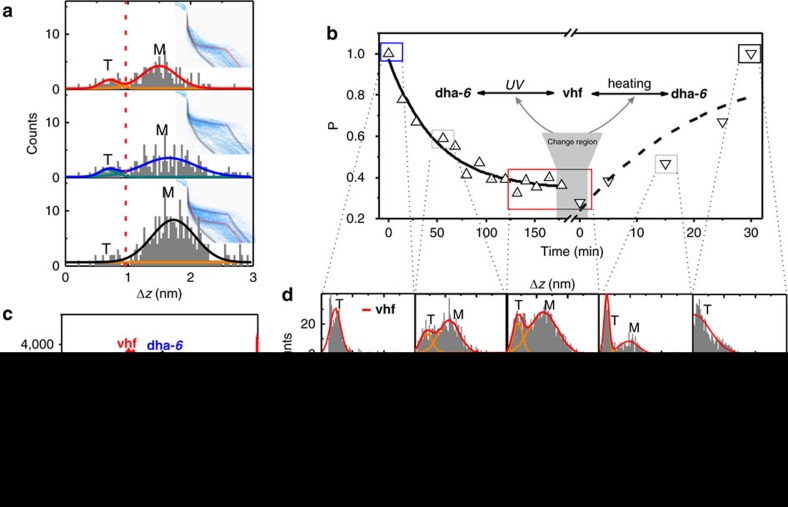
Photo-thermal reaction kinetics. (**a**) The typical relative displacement distributions of MCBJ conductance measurements with the *in situ* UV irradiation (30 min). The conductance regions of the top, middle and below section are 10^−4.5^∼10^−6^ *G*_0_ (**vhf**), 10^−3.0^∼10^−4.5^ *G*_0_ (**dha-*****6***) and whole region of 10^−3.0^∼10^−6^ *G*_0_, respectively. The Gaussian fitting is used to determine the area ratio of M and T, in which the peak marked as T is ascribed to the tunneling traces and M is for the molecular junction traces. (**b**) The **dha-*****6*** percent versus the reaction time. The upright triangle presents the data points determined from the *in situ* UV irradiation process, and fitted with the exponential function, *P*=*P*_0_+*A* × exp (−*R*_0_*t*) (black solid curve), to calculate the time decay constant. The inverted triangle shows the data points from the *ex situ* heating process, and is fitted with *P*=1−(1−*P*_0_) × exp(−*R*_0_*t*) (black dashed curve). (**c**) One-dimensional (1D) conductance histogram from different states. Blue is for the initial **dha-*****6*** state marked with blue solid box in **b**; red is for the state after *in situ* UV irradiation for 120 min, marked as red solid box in **b**; black is from the state after the heating for 30 min connected with the state marked as black solid box in **b**. The overlapping of blue and black curves suggests a highly reversible **dha-*****6***←**vhf** conversion. Due to the limited time for recording during the *in situ* UV-MCBJ measurements, the numbers of conductance curves for the statistical analysis are limited to around 500. (**d**) Plateau distribution of the break junctions from different reaction times connected to **b**. The conductance region of **vhf** is 10^−4.5^∼10^−6^ *G*_0_, and that of **dha-*****6*** is 10^−3.0^∼10^−4.5^*G*_0_.

Figure 2a shows the typical relative displacement distributions of conductance from MCBJ measurements with *in situ* UV irradiation (30 min). To determine the time-dependent junction evolution, 500 conductance curves are used for the statistical analysis for each time period. Using the conductance range of 10^−3.0^∼10^−4.5^ *G*_0_ (region 1) and 10^−4.5^∼10^−6^ *G*_0_ (region 2), we are able to construct the relative displacement distribution for **dha** and **vhf**, respectively. In the relative displacement distributions^20^ shown in Fig. 2a, the Gaussian fitting of the two peak distributions suggests that the traces with molecular junction (marked with M) are typically longer than 1 nm, while the tunnelling traces without molecular plateau are typically at around 0.5 nm (marked with T). In this way, we could determine the peak area ratio of **dha-*****6*** or **vhf** junctions from the Gaussian fitting of displacement distribution of all conductance traces including the **dha-*****6*** (or **vhf**) junction traces and tunnelling traces (see Supplementary Fig. 22 for more details). Therefore, the percentage of **dha-*****6*** junctions in all molecular junctions could be determined for the further kinetics analysis.

We use the combination of the *in situ* UV irradiation and conductance measurements to investigate the photo-conversion kinetics. The typical stretching distance distributions are presented in Fig. 2d, which are constructed from different states during the conversion process. The percentage of **dha-*****6***, along with the irradiation time, is plotted in Fig. 2b, showing that the **vhf** content reached saturation after 120 min of UV irradiation. The data (upright triangles) were fitted with the exponential function, *P*=*P*_0_+*A* × exp(−*R*_0_*t*), where *P*_0_ is the final value of the percentage, *R*_0_ is the decay constant of the best-fit curve with units of time^−1^ used for the calculation of time constant by taking the inverse. Therefore, the time constant for **dha-*****6***→**vhf** photoreaction is calculated as 45±5 min, which is higher than the previous results in the solution (less than 5 min)^22^,^23^. Two factors could be responsible for the longer time to equilibrate. Firstly, during the UV irradiation, heating caused by the light is inevitable, which leads to the back reaction (**vhf**→**dha-*****6***). More importantly, in the molecular junction, a localized surface plasmon resonance will form in nano-gaps (1.5–3 nm) between two Au electrodes under bias potential, leading to a large buildup of photon intensity and high concentration of energetic electrons^11^,^24^. During the *in situ* UV-MCBJ measurements, the localized surface plasmon resonance excitation effect could compete with the absorption of the photon from the irradiation to accelerate the back reaction. Figure 2b also illustrates that there is no complete conversion from **dha-*****6*** to **vhf**, that is, we see the decay curve plateau at just under 40% **dha-*****6***. We therefore speculate that the heating effect from irradiation and plasmon non-radiative decay must increase the back reaction rate under the *in situ* UV-MCBJ measurements. A consequence of this is that longer irradiation cannot further decrease the percentage of **dha-*****6***. It also illustrates that we could not obtain the complete conversion from **dha-*****6*** to **vhf**. Therefore, the heating effect from irradiation and plasmon non-radiative decay could increase the back reaction rate under the *in situ* UV-MCBJ measurements. It is also confirmed by the experiments with longer time of UV irradiation (Supplementary Fig. 23).

To understand the reversible ring-closing process, the *ex situ* heating of ∼60 °C was applied. As plotted in Fig. 2b (inverted triangle), an increasing percentage of **dha-*****6*** with heating time was observed and the **vhf** fully switched back to **dha-*****6*** (noted as **dha-*****6*****′** below for clarity) in 30 min. Furthermore, the function of *P*=1−(1−*P*_0_) × exp(−*R*_0_*t*) was applied for the fitting of the thermal process, and the disagreement between the fitting and the experiments suggests that the constraints of the junction may alter the kinetics of the back reaction. The 1D histograms from three states, the initial **dha-*****6***, **vhf** after *in situ* UV irradiation for 120 min, and the **dha-*****6*****′** after heating 30 min from **vhf** state, are constructed and displayed in Fig. 2c. It is shown that the conductance peak of **dha-*****6*****′** overlaps perfectly with **dha-*****6***, suggesting a highly reversible **dha-*****6***←**vhf** conversion.

### Photo/thermal conversion of the isomer

**Figure 3:**
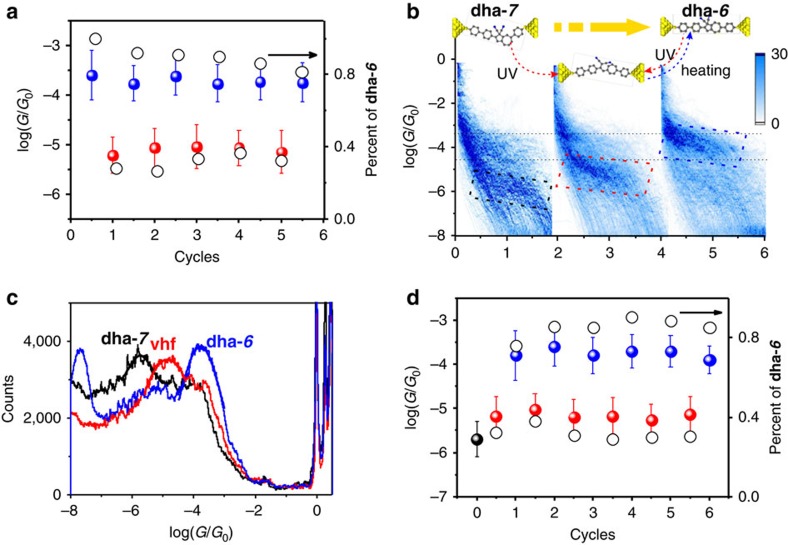
Photo/thermal conversion of the isomer. (**a**) Plot of reversible conversion of **dha-*****6***←**vhf** in six cycles. The solid circle means the conductance, related to left scale, while the hollow circle is the percentage of **dha-*****6***, related to right scale. (**b**) Schematic and 2D conductance–displacement histograms for the conversion of **dha-*****7*** with the treatments by UV irradiation and heating. (**c**) One-dimensional (1D) conductance histogram from different states. The black is from the initial **dha-*****7*** state, the red is for the state after UV irradiation to **vhf** and the blue is from the conductance measurements for solution after heating process. (**d**) Plot of conductance switch cycles starting from **dha-*****7***. The error bars of the conductance are determined from the conductance analysis based on Gaussian Function (s.d.). The solid circle means the conductance, related to left scale, while the hollow circle is the percentage of **dha-*****6***, related to right scale.

For the conversion of **dha-*****6***←**vhf,** the reversible switching cycles are performed sequentially. We demonstrate that there is no significant conversion attenuation in conductance ratio; thus, *G*_**dha-*****6***_***/**G*_**vhf**_ remained approximately 1.4, as shown in Fig. 3a. Therefore, **dha-*****6***←**vhf** can be considered as a highly promising single-molecule switch. However, the conversion ratio of **dha-*****6*** shows slight attenuation from the analysis of plateau distribution, i.e., we see slightly less **dha-*****6*** after every switching cycle. This may be due to the heat accumulation in the nano-gap regime, which significantly shifts the reaction equilibrium of the **dha-*****6***←**vhf** conversion. Together, these two results suggest that while two clear, reversible conductance states are observable at the single-molecule level, incomplete conversion might complicate the operation of a device utilizing an ensemble of molecules, and further work is required to optimize the percentage conversion in a junction.

**Figure 4:**
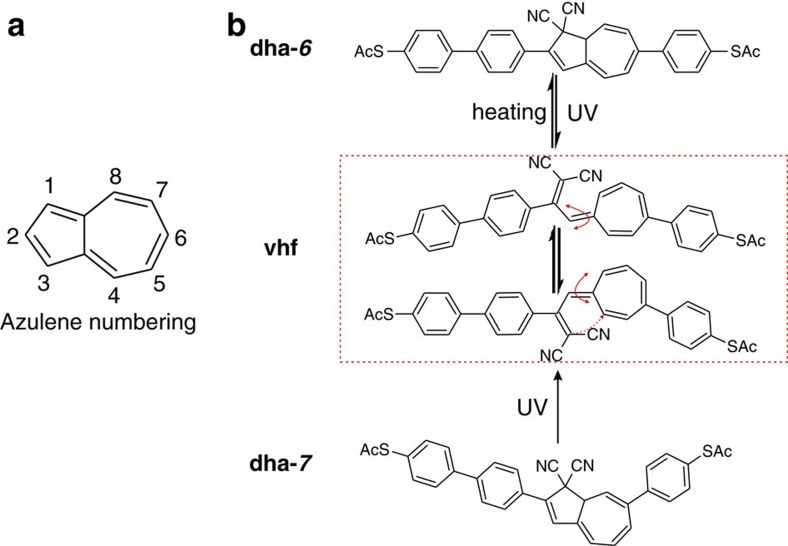
The structure of the molecules studied in this work. (**a**) The numbering used around azulene. (**b**) The molecular structures of the systems studied and the conversions observed in junction. The system can switch among **dha-*****6***, **vhf** and **dha-*****7***, where the numbers 6 and 7 refer to the position of anchoring group substitution in the seven-membered ring of **dha**.

As introduced in Fig. 4, switching between **vhf** and **dha-*****6*** is not the only possible reaction for this system as the reverse reaction can, in principle, lead to **dha-*****7***. As we did not see evidence of this conversion when starting from **dha-*****6***, **dha-*****7*** was prepared by chemical synthesis^25^ and taken as the precursor. During the conductance measurements, the operations of UV irradiation and thermal heating were also applied to **dha-*****7***. The 2D conductance–displacement and 1D conductance histograms from one photo-thermal cycle are shown in Figs 3b-3c. The conductance measurements demonstrated that the conductance of **dha-*****7*** (10^−5.8±0.1^ *G*_0_) is lower than that of **dha-*****6***, and it is also even lower than that of the ring-opened **vhf** state. More interestingly, in the case of the **dha-*****7***, after one photo/heating cycle, we only obtained one conductance peak, at *G*=10^−3.7±0.1^ *G*_0_, implying an almost complete conversion back to **dha-*****6***. Thus we conclude that the isomerization **dha-*****7***→ **vhf**→**dha-*****6*** occurred during the UV irradiation and heating reaction cycle. As shown in Fig. 3b, the UV irradiation triggers the ring-opening reaction and **vhf** is formed, followed by equilibration between **vhf** isomers. During the ring-closing reaction under the thermal heating, however, only **dha-*****6*** is formed. This can be seen in the 2D histogram, which exhibits an intensive conductance cloud around *G*=10^−3.7±0.1^ *G*_0_, ascribed to **dha-*****6.*** After the first complete switching cycle, almost no conductance features of **dha-*****7*** are observed, shown in Fig. 3b (right). Afterwards, six photo-thermal cycles were performed. It was clear that the conductive properties are quite similar with **dha-*****6***, including the reversibility and switching, as depicted in Fig. 3d. The conductance switched between high conductance (**dha-*****6***, 10^−3.7±0.1^*G*_0_) and low conductance (**vhf**, 10^−5.1±0.1^*G*_0_) with ratio of around 25.

While complete conversion of **dha-*****7*** to **dha-*****6*** was observed in the junction, which is probably promoted by a more favorable anchoring configuration of **dha-*****6*** or by a difference in the **vhf** to **dha** transition state dipole moments (*vide infra*), no such preference for **dha-*****6*** has been observed in solution. Irradiating a solution of **dha-*****7*** in CD_3_CN at 365 nm for *ca.* 2 h gave a mixture of *E*/*Z* isomeric **vhf**s according to ^1^H-NMR spectroscopy (see Supplementary Information). After 2 days in the dark, the **vhf**s underwent conversion to a mixture of **dha-*****7*** and **dha-*****6*** in a ratio of 4:3. Isomerization was also confirmed by UV–Vis absorption spectra in solution. Thus, subjecting a pristine sample of either **dha-*****6*** (*λ*_max_ 393 nm) or **dha-*****7*** (*λ*_max_ 372 nm) to one opening-closure cycle in CH_3_CN gave a mixture of isomers indicated by an intermediate absorption maximum at *ca.* 380 nm.

### Density functional theory calculations

**Figure 5:**
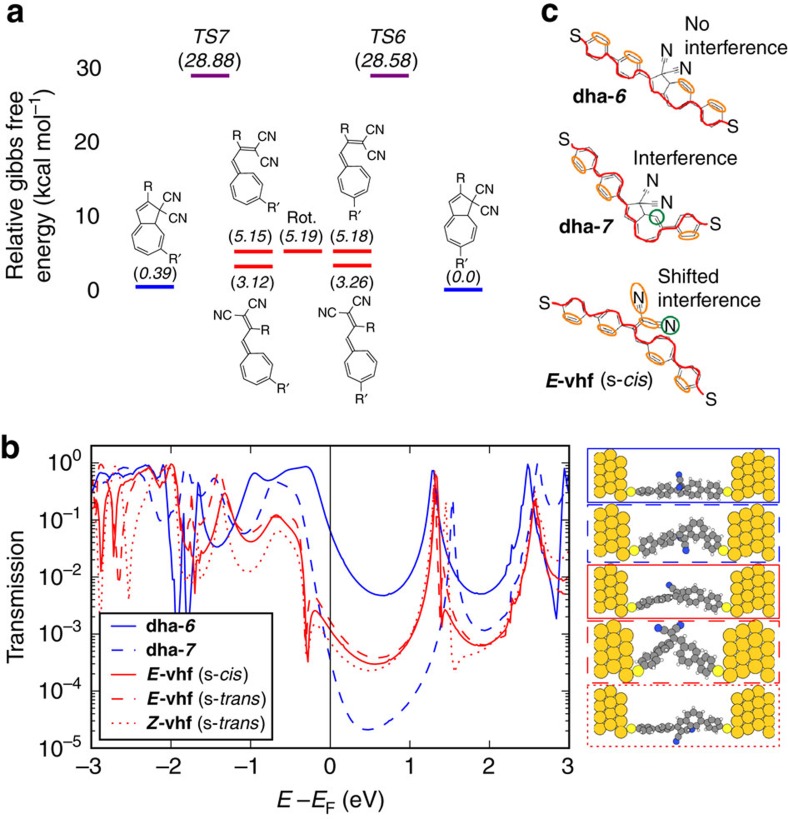
Calculated energy and transport properties. (**a**) Potential free energy profile of the **dha** and **vhf** species in acetonitrile solution. (**b**) Calculated transmissions for structures are shown on the right. Zero-bias conductance indicates *G*(**dha-*****6***)>*G*(**vhf**)>*G*(**dha-*****7***), in agreement with experiment. (**c**) Example diagrams. No interference is expected at the Fermi energy for **dha-*6*** because all diagrams without onsite loops have the same sign. For **dha-*****7***, all diagrams contain the same onsite loop so destructive interference is expected at the Fermi energy. For **vhf**, diagrams with onsite loops on N shift the interference away from the Fermi energy.

In an effort to gain insight into the reaction kinetics of the switch and the discrepancy between the behaviours in solution and junction in regard to isomerization, we investigated the potential energy surface connecting the **vhf** and **dha** isomers using density functional theory calculations. Information on the details is given in the Supplementary Information. The energy profile of the system (employing acetonitrile as solvent) is shown in Fig. 5a. We find that the free energies of **dha-*****6*** and **dha-*****7*** are very similar which is in good agreement with the outcome from experimental solution studies. The **vhf** isomers are connected to the **dha** species via transition states with similar energy barriers exceeding 20 kcal mol^−1^. The barriers for conversion between **vhf** isomers are much lower and we can therefore assume chemical equilibrium between the **vhf** isomers at all times in solution. Since the conversion from **vhf** to either **dha** is thermally activated, it is highly sensitive to the barrier heights. Any asymmetry in the barrier height would therefore lead to the promotion of one **dha** isomer over the other before chemical equilibrium is reached. The agreement between the experimental and theoretical solution studies suggests that it is something peculiar to the junction environment that leads to the switching asymmetry. One notable difference between metal electrodes and solution is the ability of electrodes to polarize and screen charges, thereby stabilizing polar/charged systems. We find that the dipole moment of the transition state of ***Z*-vhf**→**dha-*****7*** is slightly smaller than for ***E*-vhf→dha-*****6***. We therefore speculate that screening of the dipole moment from electrons in the leads or enhanced electric fields associated with the Au nano-gap might promote the local formation of **dha-*****6*** relative to **dha-*****7***. Since spontaneous ring-opening should occur at time-scales much longer than the experiment, this would explain why **dha-*****6*** is preferentially observed in the break-junction experiment.

For comparing the energies of the two molecules **dha-*****6*** and **dha-*****7*** when placed in a junction (ensuring interactions between molecule and metal leads), we performed combined QM/MM calculations^26^ using the program Dalton QCP^27^. The gold electrodes are built as two hemispheres from a fcc unit cell and described using molecular mechanics. Each gold cluster consists of 262 Au atoms, where each gold atom is assigned a polarizability of 31.04 a.u. The molecule faces the [111] surface with the axis through the two sulfur atoms oriented perpendicular to the gold surface. The density functional theory geometry-optimized molecules were inserted between the two gold clusters and then single point energy calculations were performed (CAM-B3LYP//cc-pVDZ). Interestingly, we find that **dha-*****6*** is more stable than **dha-*****7*** by 3.04 kcal mol^−1^ in the junction, while the difference was only 0.39 kcal mol^−1^ for the molecules in solution (acetonitrile). A smaller difference between the reactive s-*cis **Z*****-vhf** and s-*cis **E*****-vhf** isomers was obtained, with the ***Z*****-vhf** being 0.72 kcal mol^−1^ more stable than the ***E*****-vhf**. Although the slightly more energetic ***E*****-vhf** is the precursor for **dha-*****6***, it pays off overall from a thermodynamic point of view to form **dha-*****6*** in the junction. We were not able to perform reliable TS calculations in the junction in order to ascertain whether the kinetics of ring closure also agrees with experiment. Nevertheless, we have previously observed that the larger the **dha**−**vhf** energy difference, the faster is the **vhf**-to-**dha** conversion^27^,^28^,^29^.

**Figure 6:**
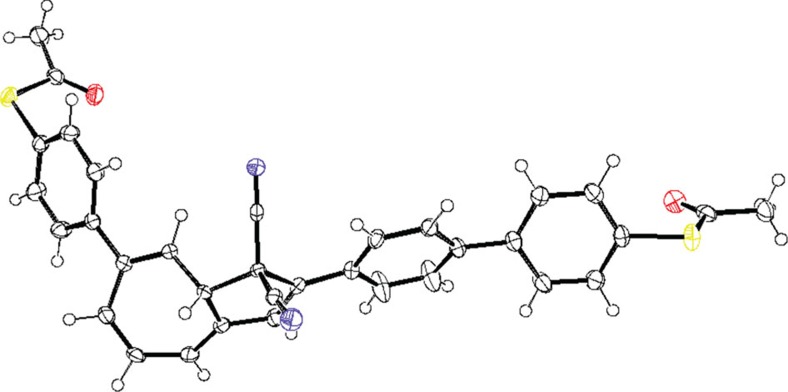
X-Ray crystal structure. Molecular structure of **dha-*****7*** with displacement ellipsoids of 50% for non H-atoms (CCDC 1501743); red: oxygen; yellow: sulfur; blue: nitrogen. Crystals were grown from dichloromethane and heptane.

Despite the chemical similarities of **dha-*****6***, **vhf** and **dha-*****7***, their calculated transport properties are vastly different and followed the trend **dha-*****6***>**vhf**>**dha-*****7*** in the experiments. A theoretical analysis using a diagrammatic approach first used by Markussen *et al*.^30^,^31^ combined with density functional theory transport calculations shows that **dha-*****6*** has the highest conductance because it is almost planar and does not show destructive interference (Fig. 5b). Conversely, **dha-*****7*** and **vhf** are bent and show destructive interference. The difference between these two systems results from the different energies at which the interference feature occurs. The diagrammatic description predicts no destructive interference for **dha-*****6*** because it is possible to pair up all the non-traversed π-orbitals along the path connecting the electrodes for these molecules. However, this is not possible for **dha-*****7*** and **vhf** as illustrated in Fig. 5c. In **dha-*****7***, the anchoring group is placed at C7, which causes significant deviation of the *π*-system from planarity as also confirmed by X-ray crystallographic analysis (Fig. 6). Moreover, the cyano groups are electronically decoupled from the rest of the π-system by saturated bonds and destructive interference therefore occurs in the middle of the band gap. This decoupling is not present in **vhf** and the anti-resonance is therefore shifted away from the middle of the band gap due to the electron-accepting character of CN. The calculated features of **dha-*****6*** and **dha-*****7*** are in line with their different optical properties determined experimentally (*vide supra*): **dha-*****6*** exhibits a red-shifted longest-wavelength absorption maximum relative to **dha-*****7***.

## Discussion

To conclude, we have developed an approach to detect the photo-thermal reaction of **dha**/**vhf** system using the single-molecule break junction technique. We have demonstrated that this technique is able to track the photo-thermal reaction, and to evaluate the reversibility and multiple switching possibilities during the reaction using the well-distinguished conductance states of single-molecule **dha**/**vhf** junctions. The combined transport calculations suggest that these distinct conductance states were ascribed to the introduction and shifting of interference features in the molecular transmission.

More importantly, beyond simply the application as a molecular switch, this study raises questions about tuning reaction kinetics, barriers and ultimately product ratios in the novel environment of molecular junctions. While we saw clear and reversible switching, the product ratio obtained does not correspond with that observed in solution studies. It remains a question for future work whether the product ratios can be further tuned by junction engineering or if this principle can be employed to tune product ratios for other reactions.

## Methods

### Single-molecule conductance measurement

Conductance measurements were performed using the MCBJ technique with a home-built MCBJ setup^19^,^20^. To perform the conductance measurement, the solution contains 0.2 mM target molecules in mixture solvent of THF:Mesitylene (TMB)=1:4. A blank experiment of solvent without target molecule is presented in Supplementary Fig. 21.

### UV irradiation and heating experiment

To have the *in situ* UV irradiation experiment, one UV LED at 365 nm is built with the power of 300 μW cm^−2^. The home-built UV LED was put on the top of the solution with a distance of ∼1 cm. As for the photo-conversion kinetic studies, the UV LED were turned on during the conductance measurements. In the *ex situ* heating process, the solution was put in a water bath with constant temperature (65 °C). After setting waiting time (0, 5, 15, 25 and 30 min), we took 200 μl solution into the liquid cell of MCBJ for conductance measurements.

### Synthesis and characterization

Synthesis and NMR characterization are provided in Supplementary Figs 1–5 and Supplementary Note 1. UV–Vis and NMR spectroscopic studies in solution are provided in Supplementary Figs 6–10 and Supplementary Note 2. Details on the theoretical work are provided in Supplementary Figs 11–20, Supplementary Table 1 and Supplementary Notes 3–6.

### Data availability

The X-ray crystallographic coordinates for the structure reported in this article have been deposited at the Cambridge Crystallographic Data Centre (CCDC), under deposition number CCDC 1501743. These data can be obtained free of charge from The Cambridge Crystallographic Data Centre via www.ccdc.cam.ac.uk/data_request/cif. The data that support the findings of this study are available from the corresponding author upon reasonable request.

## Additional information

**How to cite this article:** Huang, C. *et al*. Single-molecule detection of dihydroazulene photo-thermal reaction using break junction technique. *Nat. Commun.* **8,** 15436 doi: 10.1038/ncomms15436 (2017).

**Publisher's note**: Springer Nature remains neutral with regard to jurisdictional claims in published maps and institutional affiliations.

## Supplementary Material

Supplementary Information Supplementary figures, supplementary table and supplementary notes.

Peer review file
