# Supplementary material for: Single-molecule detection of dihydroazulene photo-thermal reaction using break junction technique
Source: Nat Commun. 2017 May 22;8:15436. doi: 10.1038/ncomms15436 (PMC5477511; doi:10.1038/ncomms15436)
Supplement: Supplementary Information — Supplementary figures, supplementary table and supplementary notes. [file ncomms15436-s1.pdf]

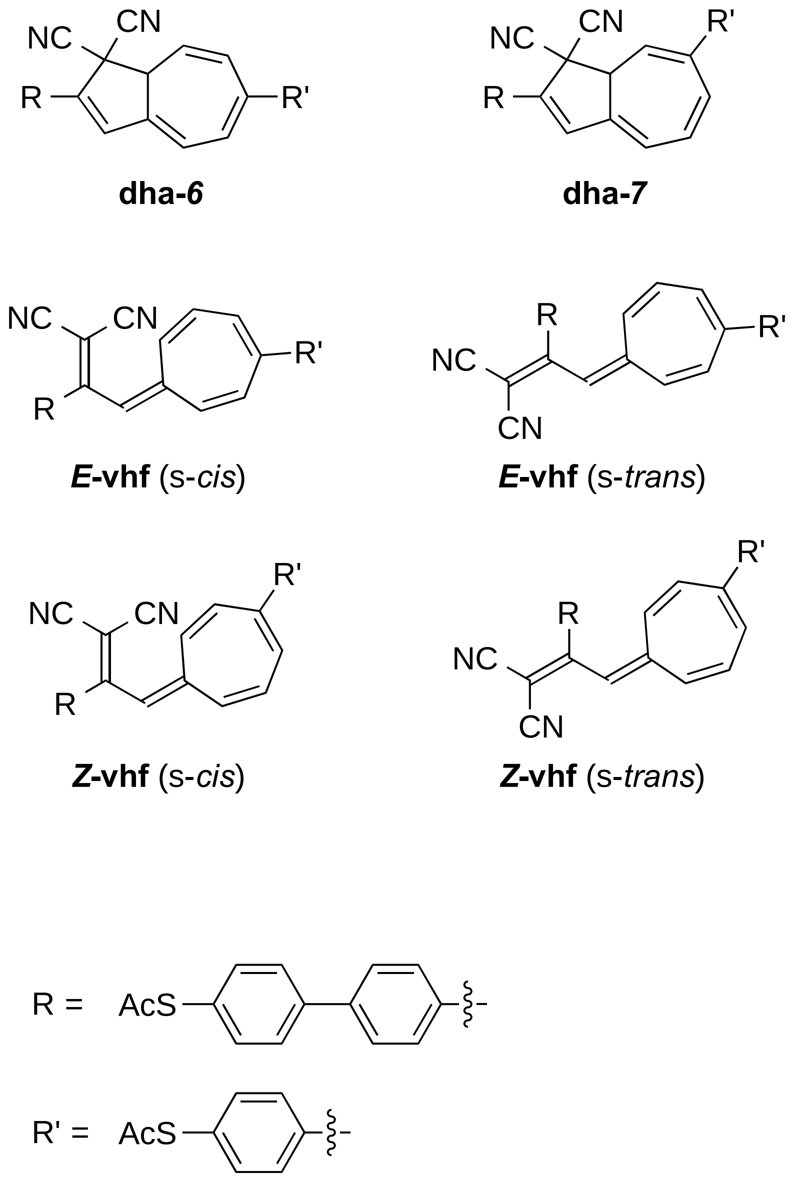

Supplementary Figure 1 | Nomenclature of compounds.

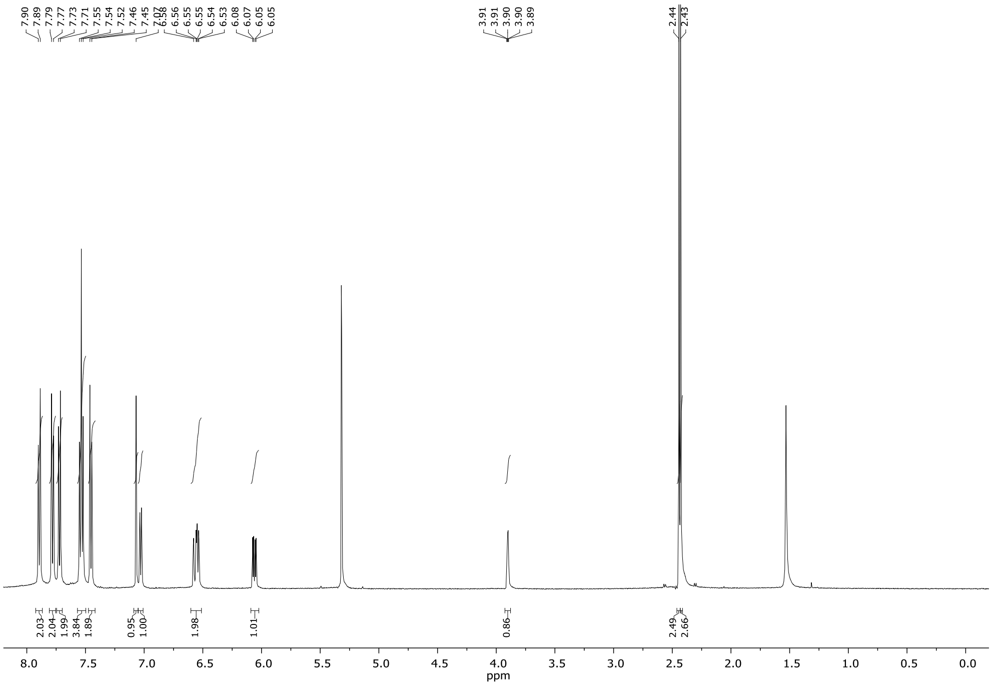

Supplementary Figure 2 | 500 MHz ^{1}H NMR spectrum of the dha-*6*.

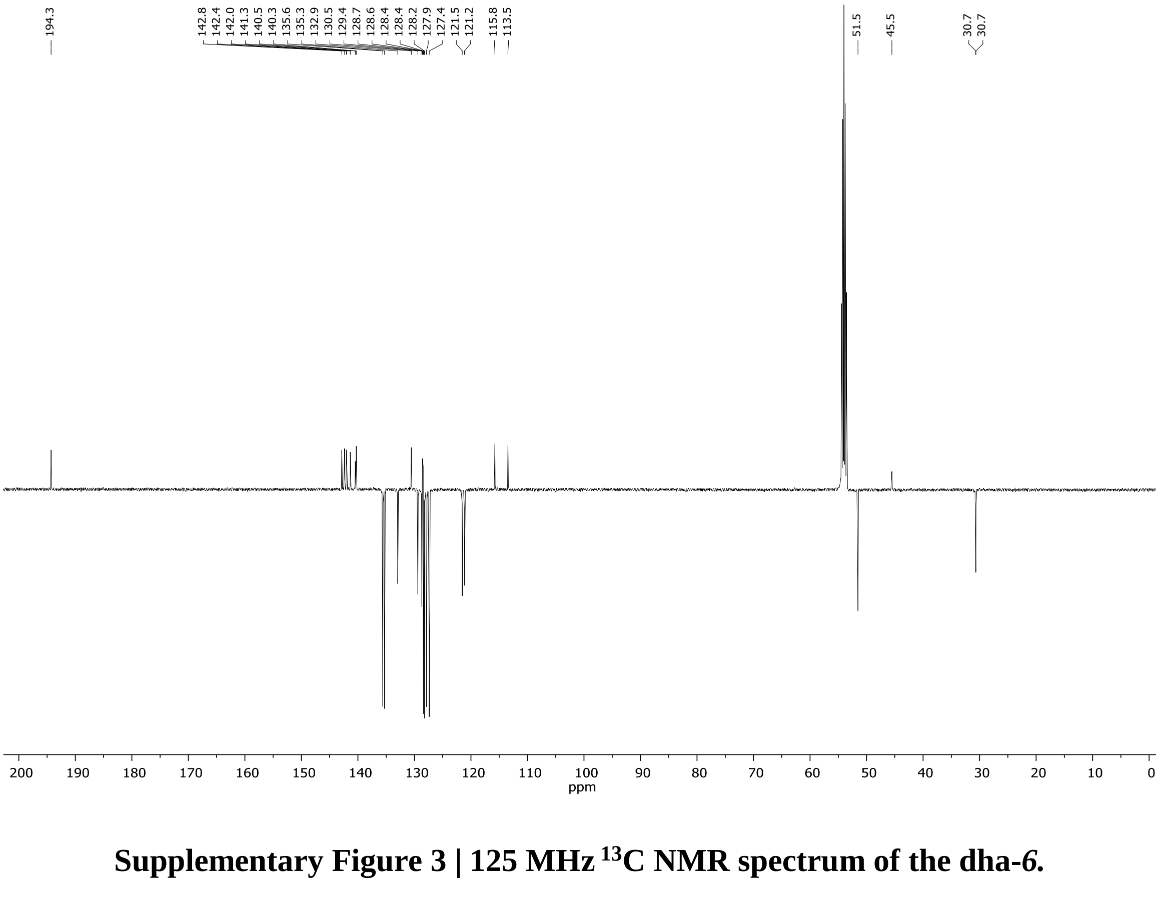

Supplementary Figure 3 | 125 MHz ^{13}C NMR spectrum of the dha-*6*.

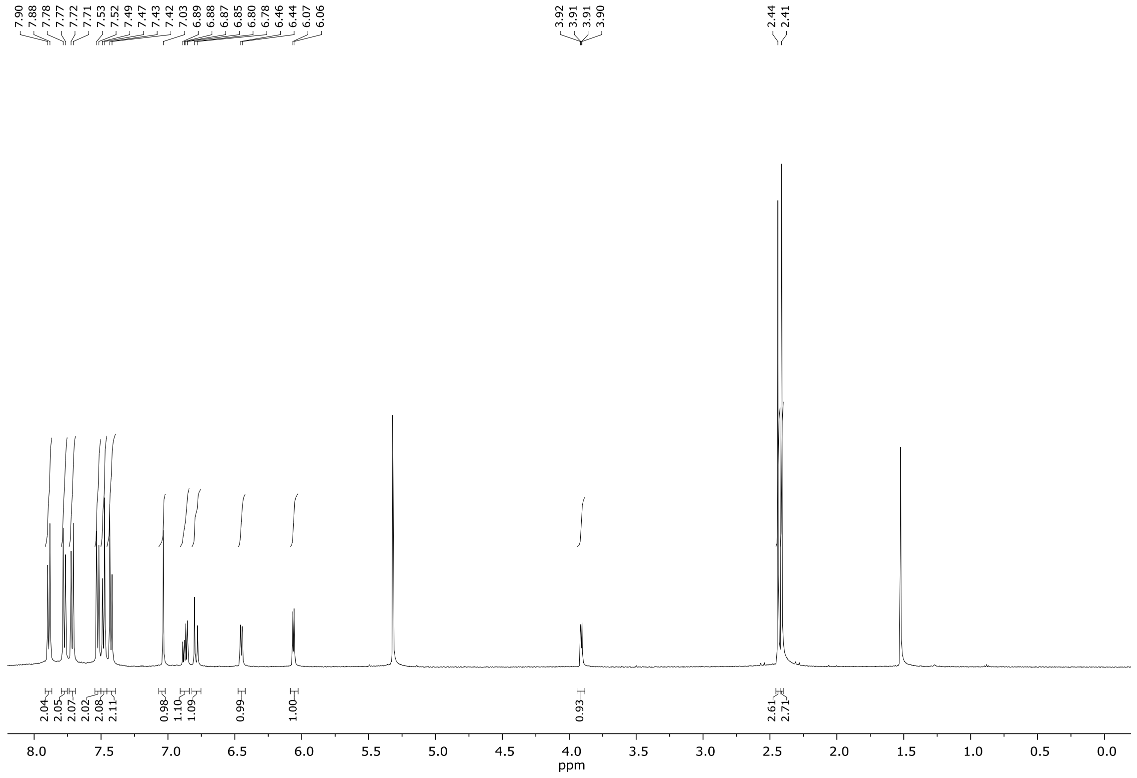

Supplementary Figure 4 | 500 MHz ^{1}H NMR spectrum of the dha-*7*.

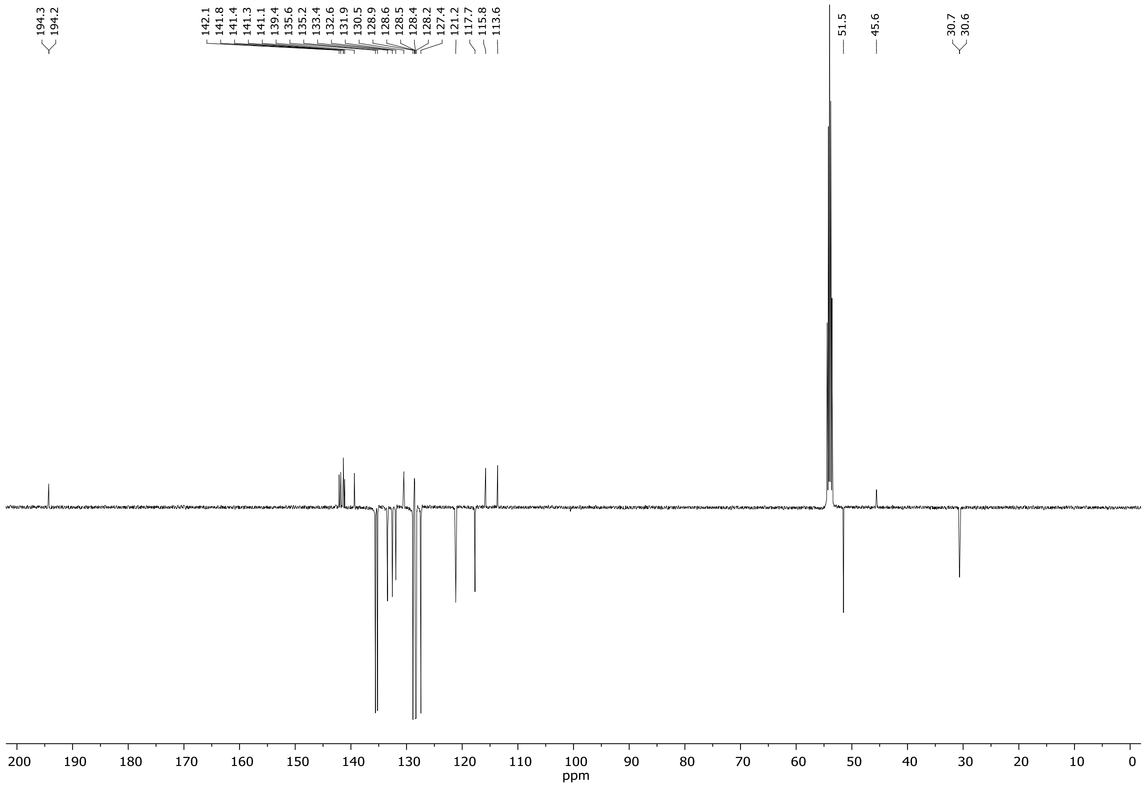

Supplementary Figure 5 | 125 MHz ^{13}C NMR spectrum of the dha-*7*.

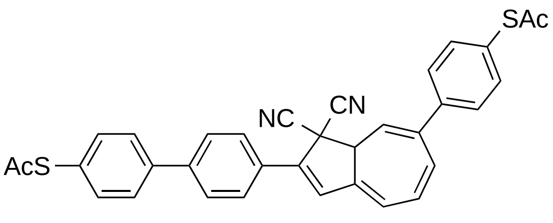

dha-7

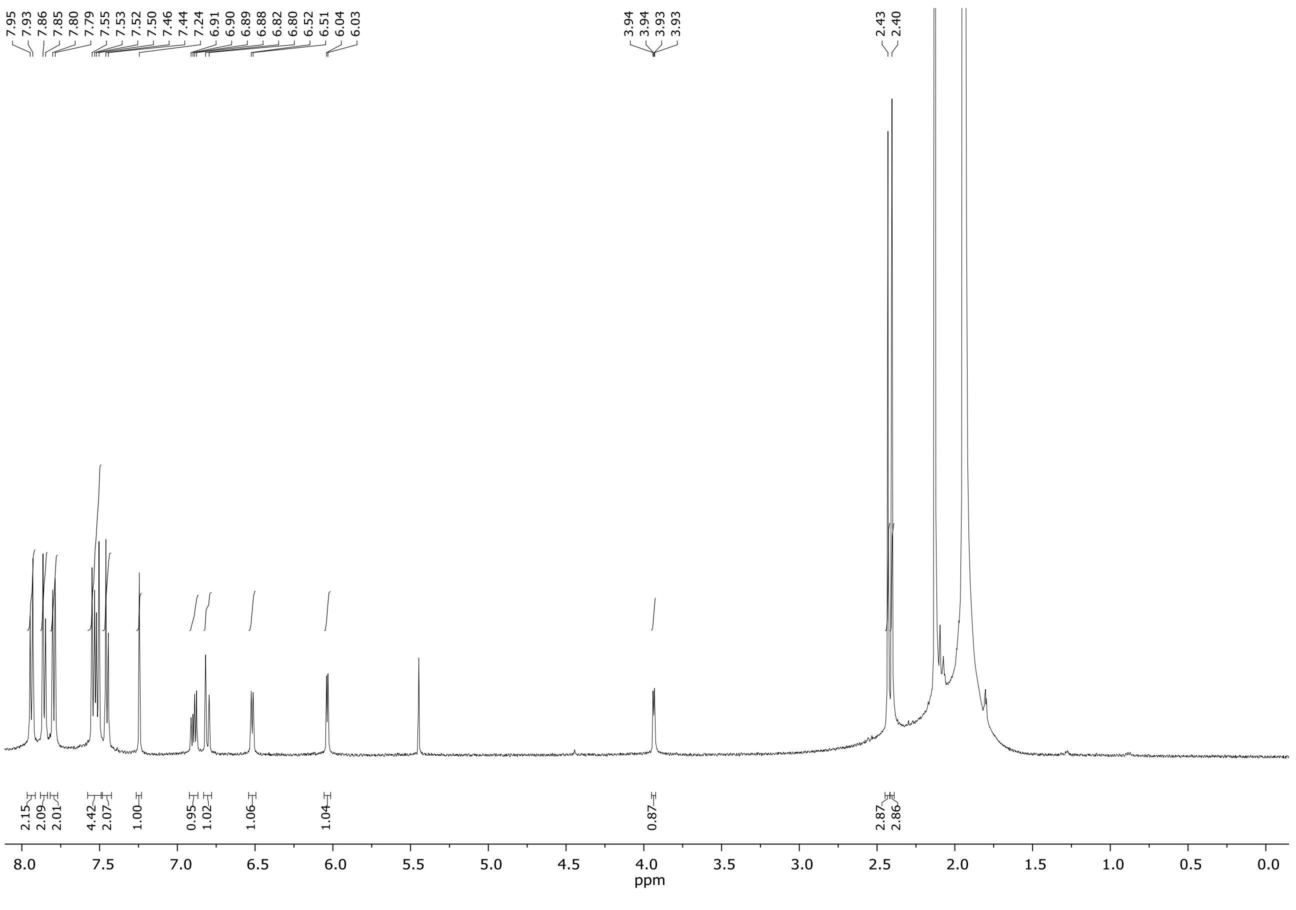

Supplementary Figure 6 | 500 MHz ^{1}H NMR spectrum of the dha-*7* in CD_3CN.

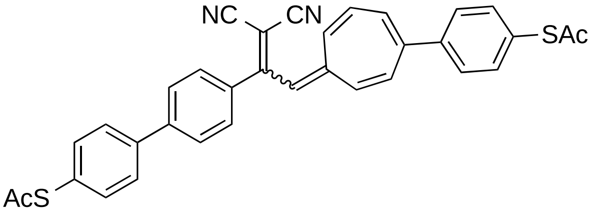

***E/Z*-vhf**

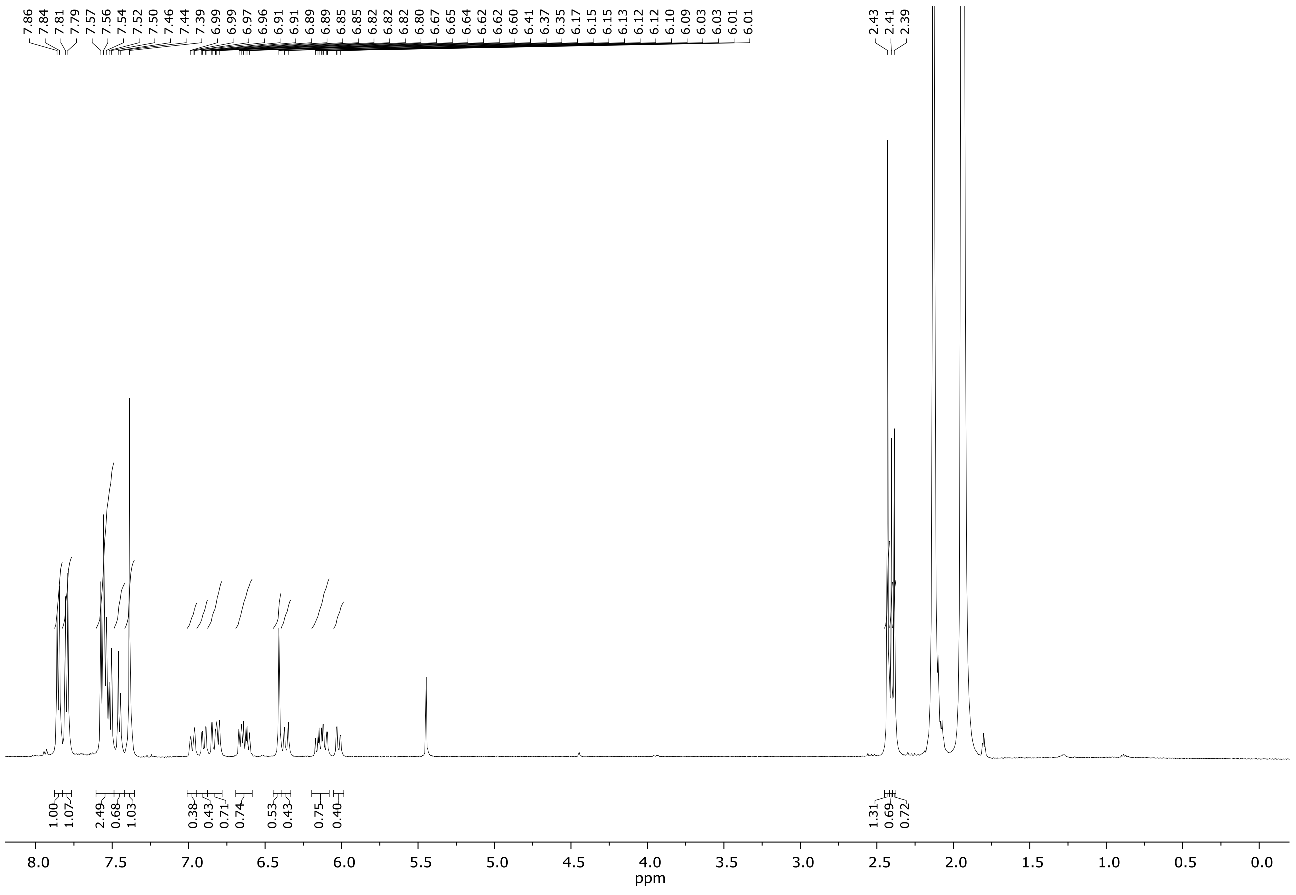

Supplementary Figure 7 | 500 MHz ^{1}H NMR spectrum of the *E/Z*-vhf in CD_3CN.

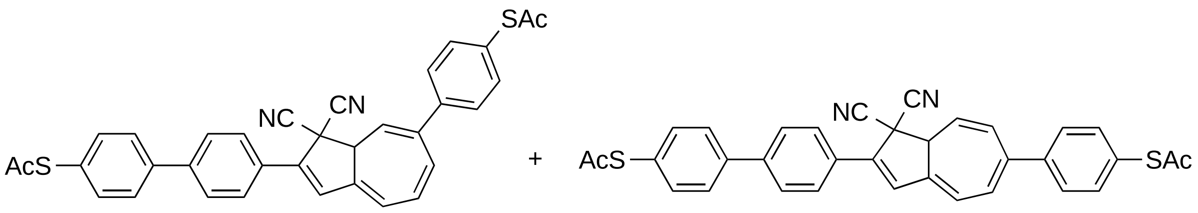

dha-*7* **dha-*6***

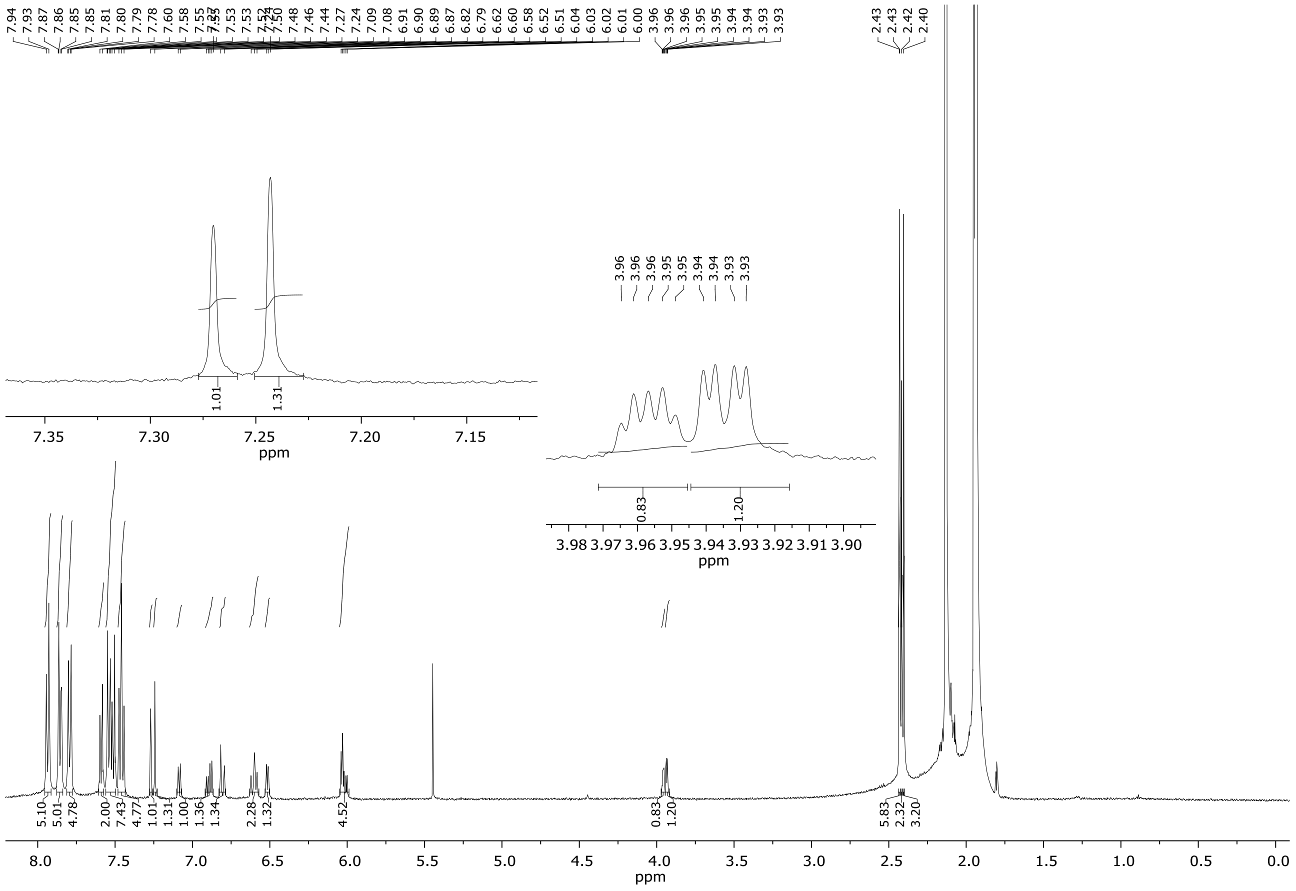

Supplementary Figure 8 | 500 MHz ^{1}H NMR spectrum of the product mixture of dha-*6* and dha-*7* in CD_3CN.

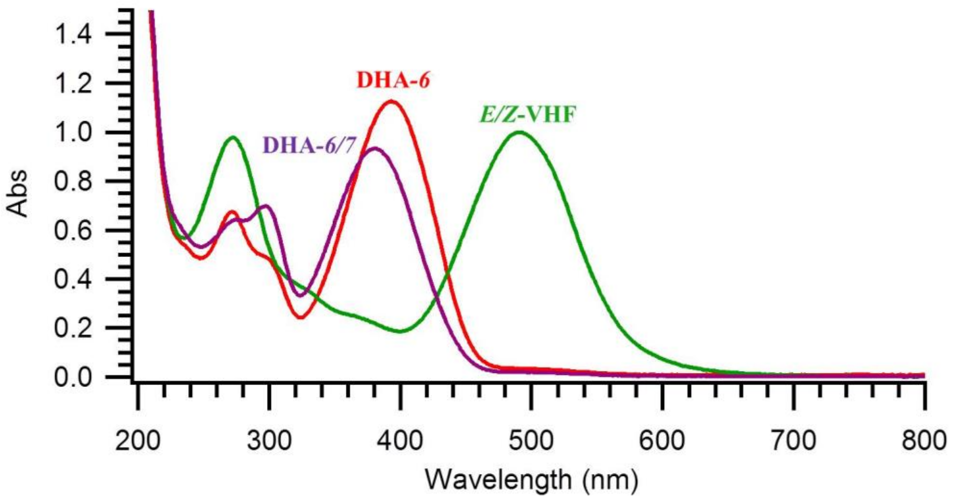

Supplementary Figure 9 | UV-Vis Absorption Spectra of DHA and VHF molecules with a pristine sample of dha-*6*. Firstly, a pristine sample of **dha-*6*** (λ_{max} = 393 nm) in CH_3CN (red) was irradiated at 365 nm to afford the ***E/Z*-vhf** (λ_{max} = 491 nm), depicted in green. Thermal ring closure at ambient temperature resulted in the formation of a mixture of **dha-*6*** and **dha-*7*** (λ_{max} = 380 nm), as shown by the magenta colored line.

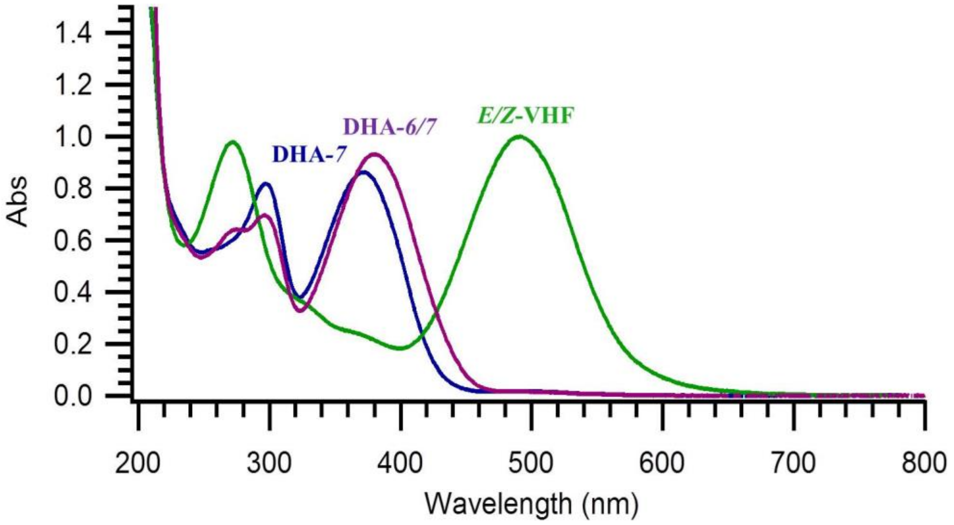

Supplementary Figure 10 | UV-Vis Absorption Spectra of DHA and VHF molecules with a pristine sample of dha-*7* Likewise, UV-vis traces, which firstly shows a solution of pure **dha-*7*** (λ_{max} = 372 nm) in CH_3CN indicated by the blue line, which was irradiated at 365 nm to afford the ***E/Z*-vhf** (λ_{max} = 491 nm), depicted in green. Thermal ring closure at ambient temperature resulted in the formation of a mixture of **dha-*6*** and **dha-*7*** (λ_{max} = 380 nm), as shown by the magenta colored line.

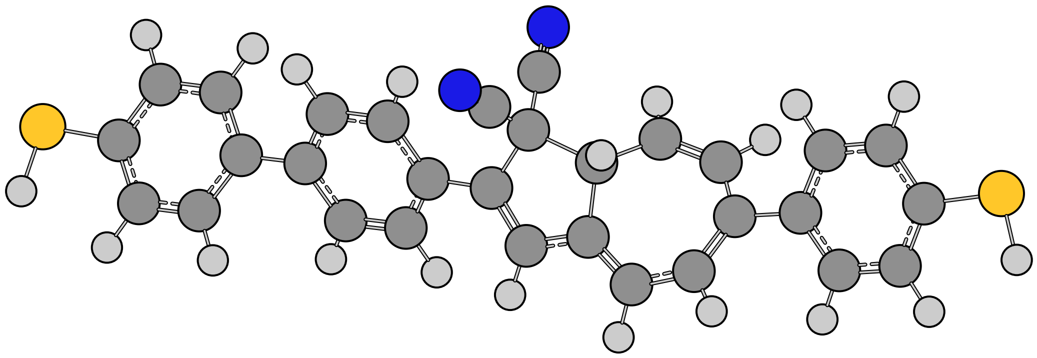

Supplementary Figure 11 | Structure of dha-*6*.

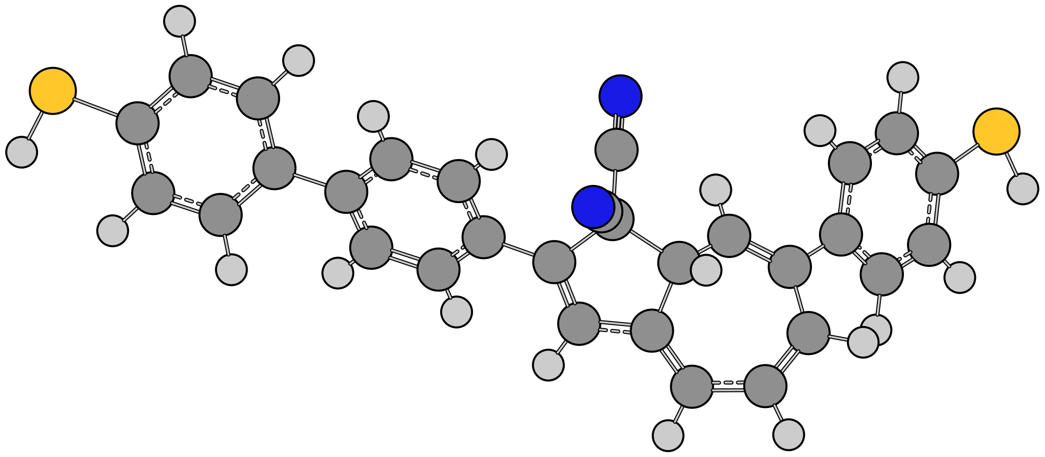

Supplementary Figure 12 | Structure of dha-*7*.

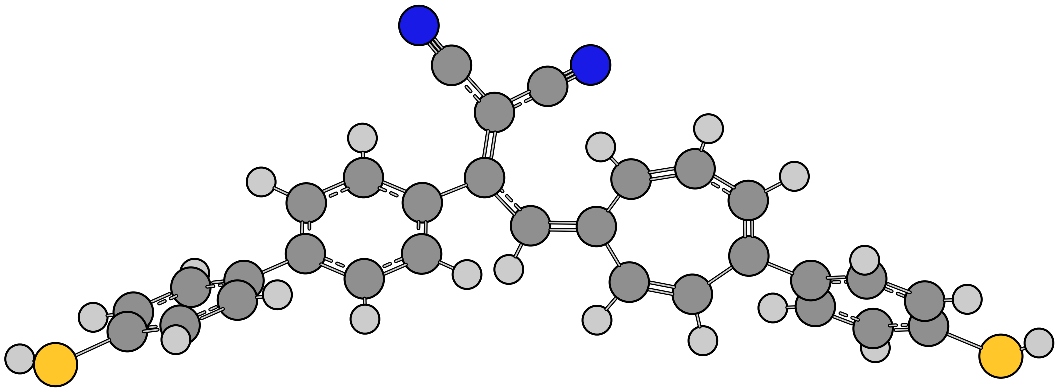

Supplementary Figure 13 | Structure of *E*-vhf (s-*cis*).

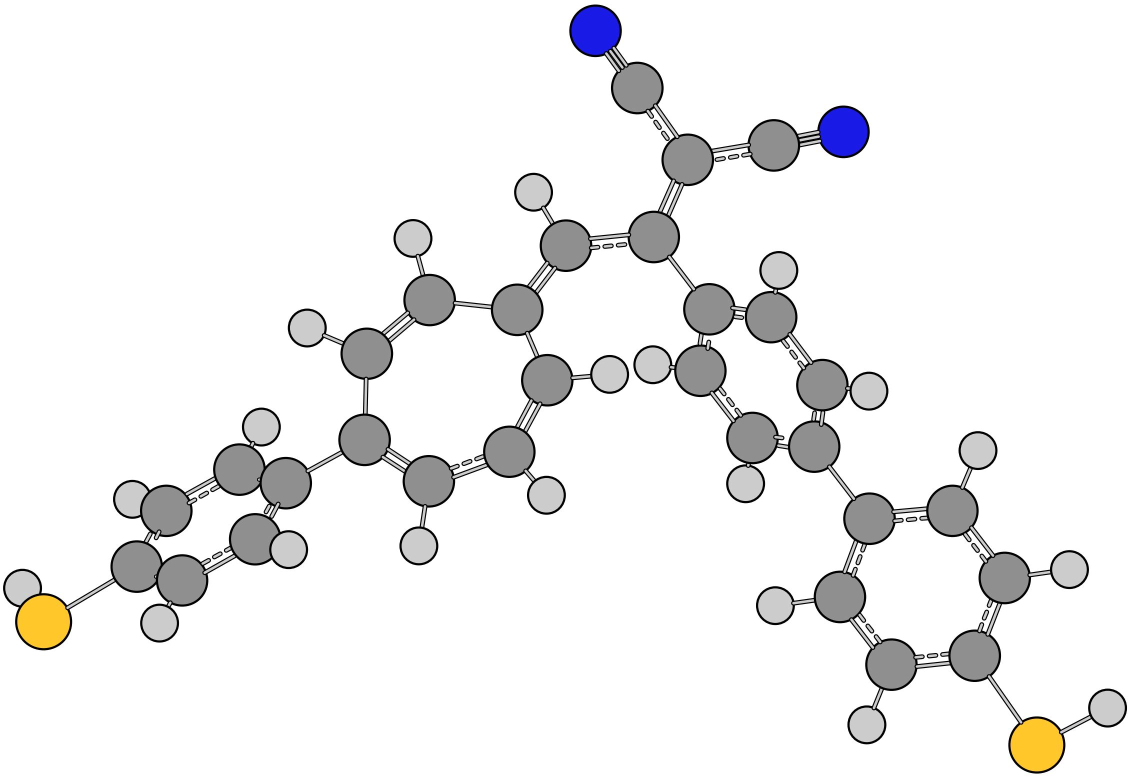

Supplementary Figure 14 | Structure of *E*-vhf (s-*trans*).

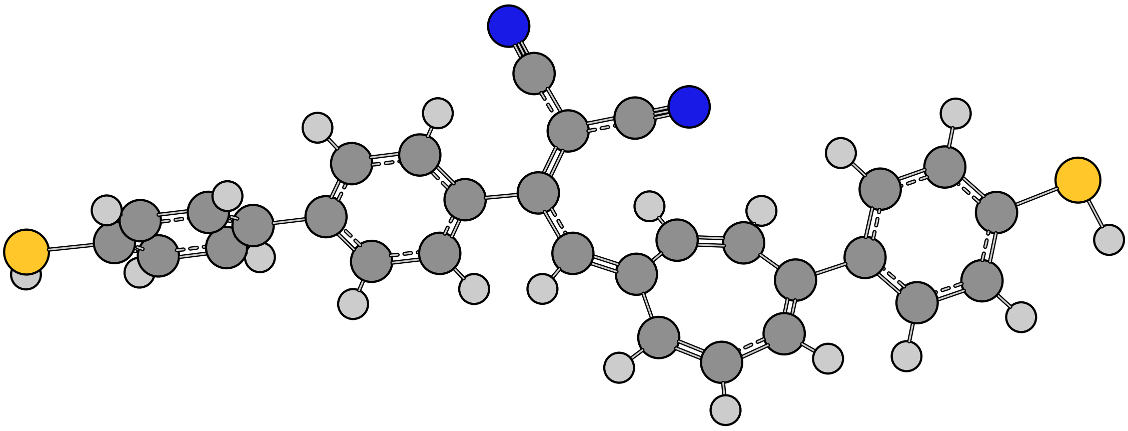

Supplementary Figure 15 | Structure of Structure of *Z*-vhf (s-*cis*).

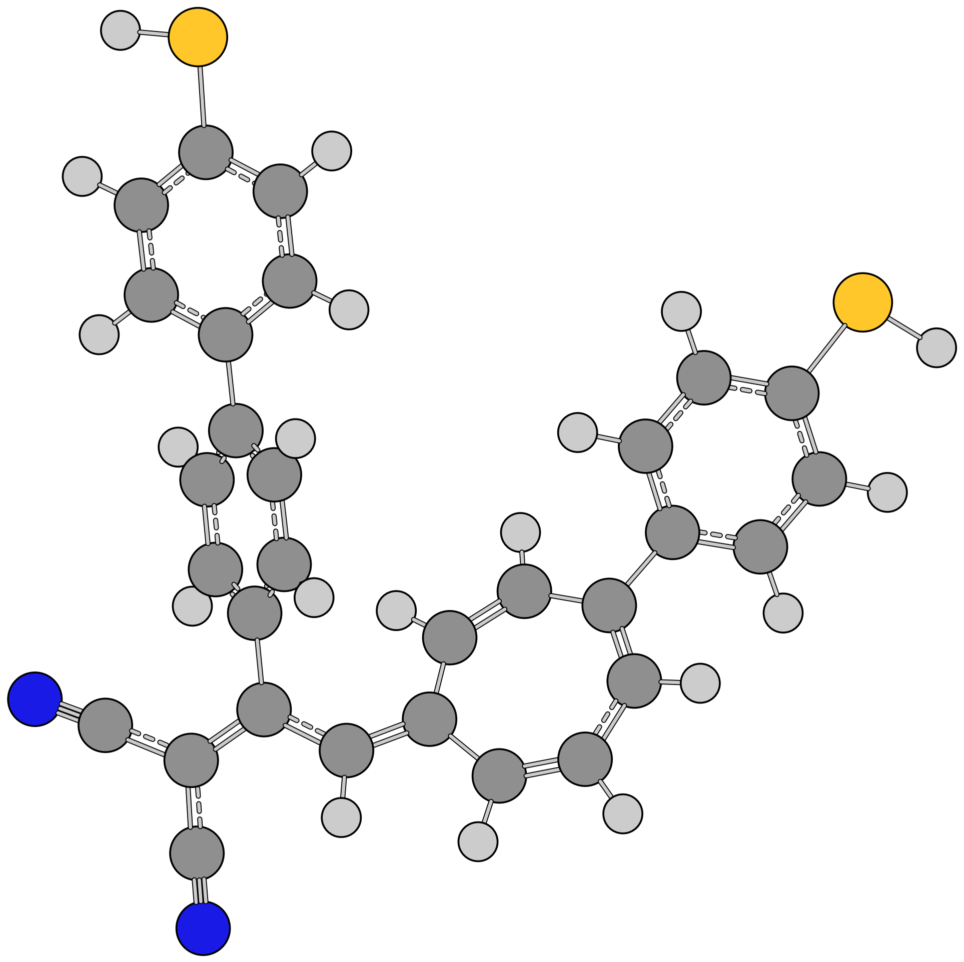

Supplementary Figure 16 | Structure of *Z*-vhf (s-*trans*).

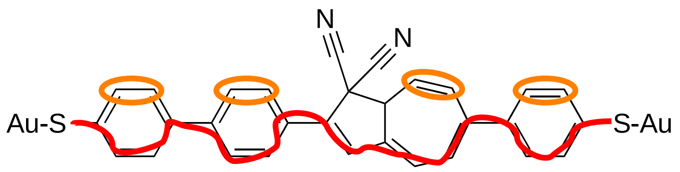

Supplementary Figure 17 | Markussen diagrams of dha-*6*. No matter which path through the π-system connects the electrodes the remaining sites can always be paired up in such a way that there are no on-site loops. This means that there is always at least one term in $det_{1N}(EI - H)$ that does not depend on E. For $E = \epsilon_C{\sim}0$ to be a root of $det_{1N}(EI - H)$ this term must be cancelled by the rest of the terms. Those terms that contain an onsite loop vanish at $E=0$ and those that do not all have the same relative sign. This means that $E=0$ cannot be a root of the polynomial and destructive interference should not occur at the Fermi energy.

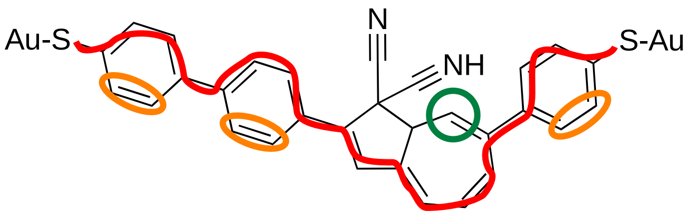

Supplementary Figure 18 | Markussen diagrams of dha-*7*: In order for a path through the π-system to connect the electrodes there is always the same isolated site that must have an onsite loop. This means that all terms contain a factor of $(E - \epsilon_C)$ and destructive interference therefore occurs at $E = \epsilon_C{\sim}0$.

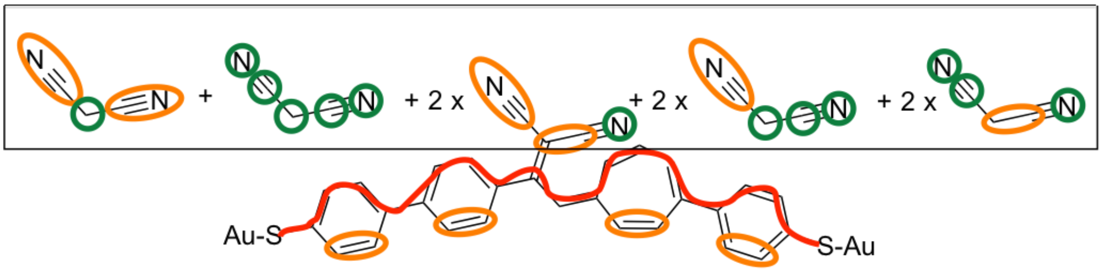

Supplementary Figure 19 | Markussen diagrams of vhf. In order for a path to connect the electrodes there is always the same isolated set of sites. It is shown all possible diagrams to be drawn for these sets of sites. Since there are an uneven number of isolated sites connected in series all terms

contain an onsite loop. ϵ_C is however not a root of the polynomial since $\epsilon_C \neq \epsilon_N$. But the on-site energies of carbon and nitrogen are similar compared to the coupling parameter so $E=0$ is almost a root. This root close to zero constitutes what we denote as a shifted interference feature and explains why the **dha-*7*** is a worse conductor than **vhf** in spite of both being cross-conjugated.

	**Relative Energies** (kcal/mol)	**Junction** (kcal/mol)	**Relative Energies** (kcal/mol)
**dha-*6***	0.00	-1292070.76	0.00
**TS6**	29.58	-	-
***E*-vhf** (s-*cis*)	5.18	-1292057.52	13.24
***Z*-vhf** (s-*cis*)	5.15	-1292058.24	12.52
**TS7**	28.88	-	-
**dha-*7***	0.39	-1292067.73	3.04
***E*-vhf** (s-*trans*)	3.26	-1292061.43	9.33
***Z*-vhf** (s-*trans*)	3.12	-1292065.47	5.29
**Rot**	5.19	-	-

Supplementary Table 1 | Energies for molecules in solvent (acetonitrile) and junction. For the calculations in solution, the molecules are end-capped with SH groups (not SAc), while they are end-capped with S in the junction. **TS6** is the transition state structure from ***E*-vhf** (s-*cis*) to **dha-*6***; **TS7** is the transition state structure from ***Z*-vhf** (s-*cis*) to **dha-*7***; **Rot** is the TS structure between *Z*- and *E*-**vhf** in s-*cis* conformations.

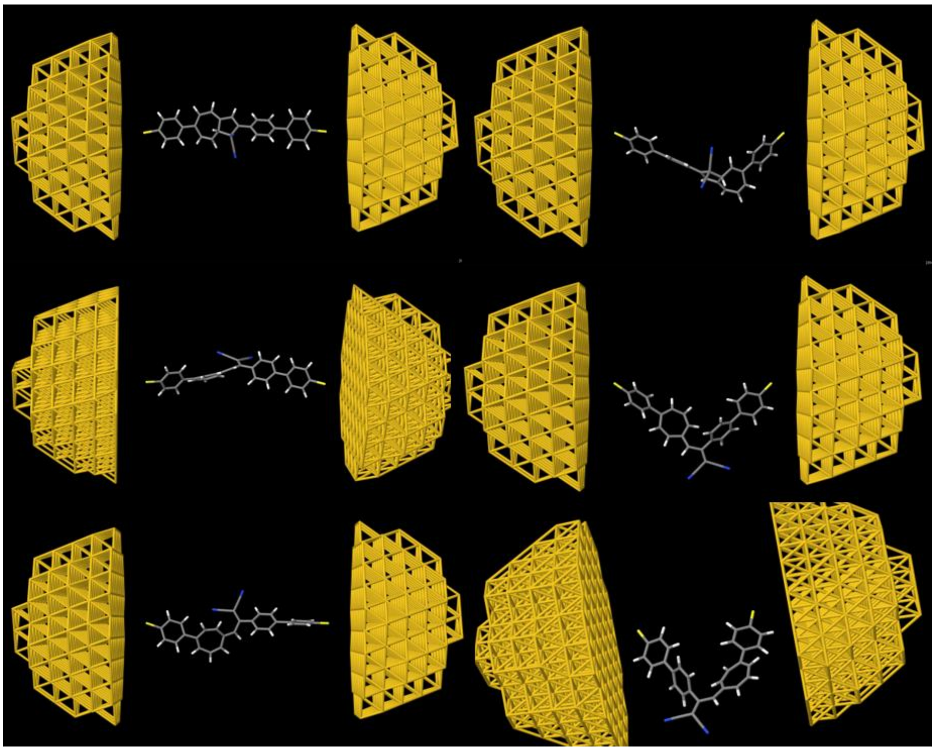

Supplementary Figure 20 | Schematic representations of the junction set-up. From top to bottom (left / right): **dha-*6* / dha-*7***, ***E*-vhf** (s-*cis* / s-*trans*), ***Z*-vhf** (s-*cis* / s-*trans*). A combined QM/MM method was used to calculate the energy of **dha-*6*** and **dha-*7*** in a junction and their **vhf** forms, ensuring the inclusion of the screening effect of the metal leads. The molecule is treated quantum mechanically by DFT using the long-range corrected functional CAM-B3LYP and the correlation consistent basis set cc-pVDZ. The molecular mechanically gold electrodes are built as two hemispheres from a fcc unit cell. Each gold cluster is consisting of 262 Au atoms, where each gold atom is assigned with a polarizability of 31.04 au. The molecule faces the [111] surface with the axis through the two sulfur atoms standing perpendicular to the gold surface.

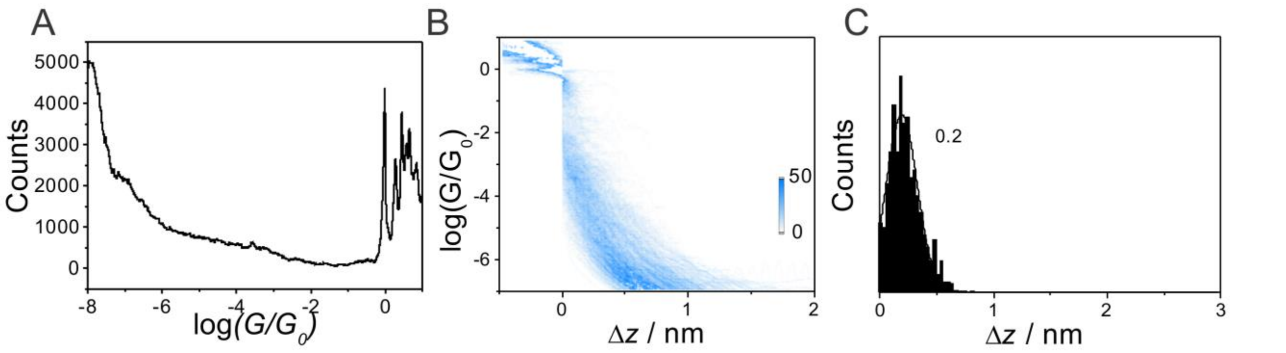

Supplementary Figure 21 | MCBJ experiment in solvent without molecules. The conductance histogram in the pure solvent without adding molecules. A: 1D conductance histogram; B: 2D

conductance histogram C: the relative displacement distribution histogram determined from the conductance range between 0.7 G_0 to 10^{-3} G_0. Clean background suggested no molecular junction are formed during the experiment with pure solvent.

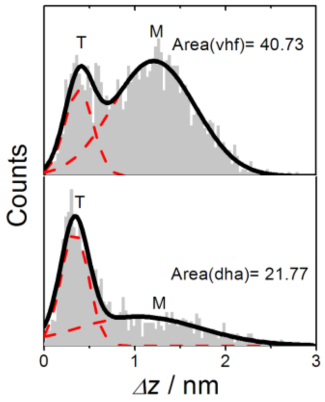

Supplementary Figure 22 | Determination of the percentage of dha-*6* junction from relative displacement distributions. Relative displacement distributions of MCBJ conductance measurements with the *in-situ* UV irradiation (120 min). The conductance regions of the top and below panel are $10^{-4.5}$ ~10^{-6} G_0 (**vhf**), $10^{-3.0}$ ~ $10^{-4.5}$ G_0 (**dha-*6***), respectively. The relative displacement distribution histogram was fitting by Gaussian function, and the peaks assigned to molecular feature are marked with M. The peak areas were integrated to determine the number of traces with specific molecular feature. Thus we could determine area ratio of **dha-*6*** is 0.34 (= 21.77/(21.77+40.73)), which is relevant to the percentage of **dha-*6*** molecules in this experimental condition. To further validate the determination of area ratio in another way, we could also count the number of traces with relative displacement longer than 0.72 nm, where 0.72 nm was determined from the cross point of the two peak fitting. We find that the number of trace for vhf is 108, and the number for **dha-*6*** is 63. the percentage of **dha-*6*** is around 0.33 (=63/(63+108)), which agree with the area ratio method we applied in this work well.

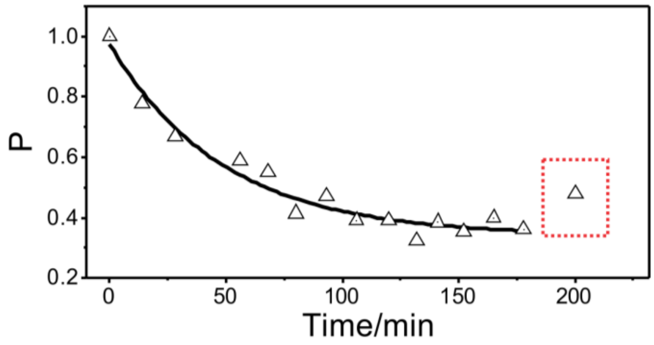

Supplementary Figure 23 | Heating induced back reaction from dha-*6* to vhf The percentage of **dha-*6*** versus the reaction time during UV irradiation. When the UV irradiation was extended to 200 min, it is clear that the percentage of **dha-*6*** increased after the equilibrium state (the red-dots block). The increasing of the back reaction during *in-situ* UV-MCBJ measurements could be responsible for the shift of the reaction equilibrium.

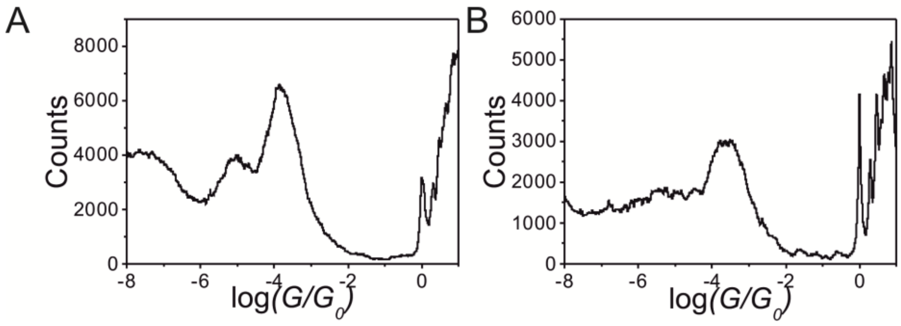

Supplementary Figure 24 | Control experiments using new MCBJ samples to compare reaction in junction and in solution A: the conductance histogram of solution (**dha-*6***) with UV irradiation (15 min) measured by a new MCBJ sample; B: the conductance histogram of solution (**vhf**) after thermal treatment (50 min) measured by a new MCBJ sample. We used a new MCBJ sample sheet to carry out the measurement of the solution after UV irradiation and thermal treatment as control measurement. We got only one conductance feature after the thermal treatment using a new MCBJ sample sheet, which could be assigned to **dha-*6***. No signal of **dha-*7*** (located at 10^{-6} G_0) was found in the control experiment. While in the solution, after thermal treatment of **vhf** solution, we got the mixture of **dha-*6*** and **dha-*7*** in UV-Vis absorption spectra, as shown in Supplementary Figure 9. The difference suggested **dha-*6*** are highly preferred in a junction configuration.

Supplementary Note 1 | Synthesis

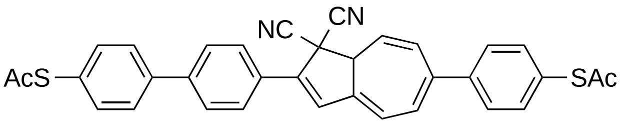

dha-*6*

S*-(4-(2-(4'-(Acetylthio)-[1,1'-biphenyl]-4-yl)-1,1-dicyano-1,8a-dihydroazulen-6-yl)phenyl) ethanethioate (dha-*6*)**; A 2:1 mixture (163 mg) of **dha-*6 and **dha-*7*** prepared by the reported procedure (Jevric, M., Broman, S. L., Nielsen, M. B., *J. Org. Chem.* **2013**, *78*, 4348-4356) was crystalized from hot toluene (30 mL) to give the **dha-*6*** as a fluffy yellow solid (71 mg). M.p. 225–227 °C; ^{1}H NMR (500 MHz, CD_2Cl_2) δ 7.89 (d, *J* = 8.4 Hz, 2H), 7.78 (d, *J* = 8.4 Hz, 2H), 7.72 (d, *J* = 8.3 Hz, 2H), 7.55-7.52 (m, 4H), 7.45 (d, *J* = 8.3 Hz, 2H), 7.07 (s, 1H), 7.03 (d, *J* = 6.8 Hz, 1H), 6.58-6.53 (m, 2H), 6.06 (dd, *J* = 10.3, 4.2 Hz, 1H), 3.90 (dt, *J* = 4.2, 2.1 Hz, 1H), 2.44 (s, 3H), 2.43 (s, 3H) ppm; ^{13}C NMR (125 MHz, CD_2Cl_2) δ 194.3, 142.8, 142.4, 142.0, 141.3, 140.5, 140.3, 135.6, 135.3, 132.9, 130.5, 129.4, 128.7, 128.6, 128.4, 128.4, 128.2, 127.9, 127.4, 121.5, 121.2, 115.8, 113.5, 51.5, 45.5, 30.7, 30.7 ppm, 1C masked.

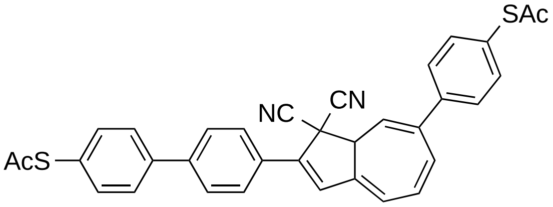

dha-*7*

***S*-(4-(2-(4'-(Acetylthio)-[1,1'-biphenyl]-4-yl)-3,3-dicyano-3,3a-dihydroazulen-5-yl)phenyl) ethanethioate (dha-*7*)**; Synthesised according to the literature procedure (Jevric, M., Broman, S. L., Nielsen, M. B., *J. Org. Chem.* **2013**, *78*, 4348-4356). ^{1}H NMR (500 MHz, CD_2Cl_2) δ 7.89 (d, *J* = 8.5 Hz, 2H), 7.77 (d, *J* = 8.5 Hz, 2H), 7.72 (d, *J* = 8.4 Hz, 2H), 7.52 (d, *J* = 8.4 Hz, 2H), 7.48 (d, *J* = 8.4 Hz, 2H), 7.43 (d, *J* = 8.4 Hz, 2H), 7.03 (s, 1H), 6.87 (dd, *J* = 11.5, 6.1 Hz, 1H), 6.79 (d, *J* = 11.5 Hz, 1H), 6.45 (br d, *J* = 6.1 Hz, 1H), 6.06 (d, *J* = 4.6 Hz, 1H), 3.91 (dd, *J* = 4.6, 1.5 Hz, 1H), 2.44 (s, 3H), 2.41 (s, 3H) ppm; ^{13}C NMR (125 MHz, CD_2Cl_2) δ 194.3, 194.2, 142.1, 141.8, 141.4, 141.3, 141.1, 139.4, 135.6, 135.2, 133.4, 132.6, 131.9, 130.5, 128.9, 128.6, 128.5, 128.4, 128.2, 127.4, 121.2, 117.7, 115.8, 113.6, 51.5, 45.6, 30.7, 30.6 ppm.

The NMR are presented in Supplementary Figure 2-5.

Supplementary Note 2 | Switching Experiments in Solution

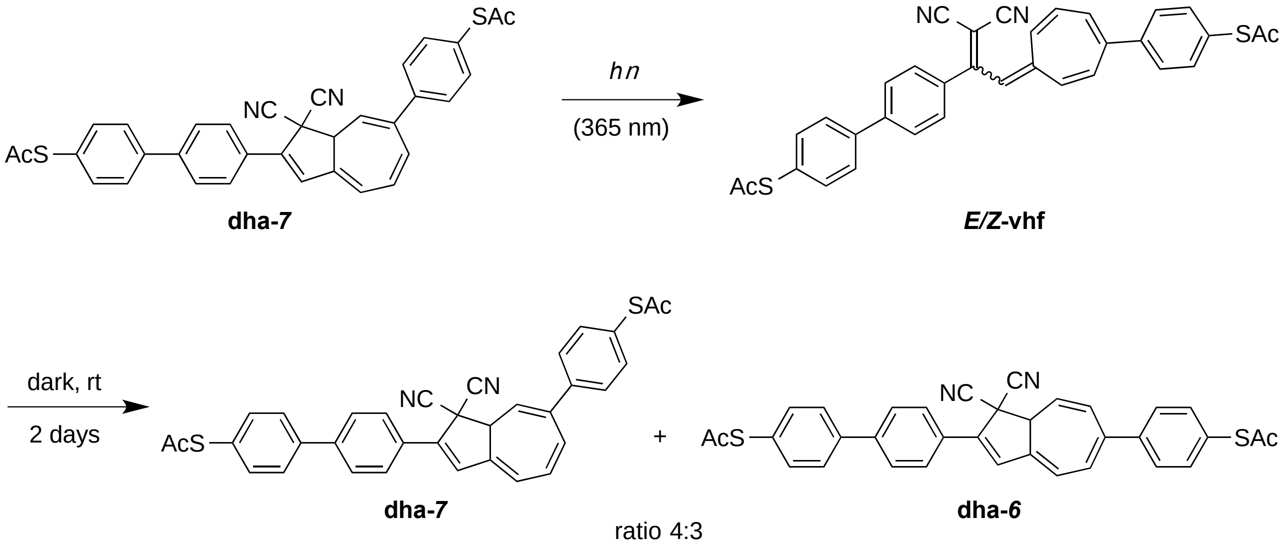

dha-*7* (1 mg) was dissolved in CD_3CN (0.75 mL) and the solution irradiated at 365 nm by the means of a TLC lamp (ca. 2 hours) until ^1H NMR determined complete ring opening to the ***E/Z*-vhf**. The sample was then kept in the dark for two days and the ^1H NMR spectrum was remeasured to give a mixture of **dha-*7*** and **dha-*6*** in a ratio of approximately 4:3.

The NMR are presented in Supplementary Figure 6-8.

Supplementary Note 3 | Reaction Kinetics Calculation

In order to understand the difference **dha-*6***: **dha-*7*** ratio in the solution study, calculations for the reaction kinetics were performed in Gaussian09 (the calculations included acetonitrile as solvent). A potential energy surface search was run on the hydrogen terminated structures of **dha-*7***, **dha-*6***, ***Z*-vhf** s-*cis*, **Z-vhf** s-*trans*, ***E*-vhf** s-*cis*, and ***E*-vhf** s-*trans* in order to find the minimum energy structures. From here, the transitions states and the rotational barrier between the two **vhf** forms were found. With DFT/CAM-B3LYP/cc-pVDZ the Gibbs free energies were calculated.

The structures obtained are presented in Supplementary Figure 11-16.

Supplementary Note 4 | Junction Calculations

We use the diagrammatic technique developed by Markussen *et al.* for model systems and validate the results using density functional theory coupled to a non-equilibrium Green's function approach.

According to the Landauer formula the transmission can be calculated as

$$T(E,V) = Tr(\Gamma_L G^r \Gamma_R G^a)$$

where G^r and G^a are the retarded and advanced Green's function and $\Gamma_{L/R}$ are calculated from the lead self energies according to $\Gamma_\alpha = i[\Sigma_\alpha^r - \Sigma_\alpha^a]$. Here Σ_α^r and Σ_α^a are the retarded and advanced selfenergies of lead α. For simple Hückel models where only site *1* couples to the left electrode with effective coupling parameter γ_L and site *N* couples to the right electrode with coupling parameter γ_R, the only non-zero element in the matrix product above is $\gamma_L \gamma_R |G_{1N}|^2$. Using Cramer's formula the matrix element can be evaluated (up to an arbitrary sign) as:

$$G_{1N} = \frac{det_{1N}(EI - H)}{\det(EI - H - \Sigma_L - \Sigma_R)}$$

For destructive interference we therefore require that $det_{1N}(EI - H) = 0$ while $\det(EI - H - \Sigma_L - \Sigma_R) \neq 0$.

$det_{1N}(EI - H)$ is a polynomial in E with roots where the transmission goes to zero. It can be evaluated diagrammatically by drawing all diagrams following the set of rules:

- In each diagram a continuous path must connect the sites *1* and *N*.
- All internal sites must either have one ingoing and one outgoing path or have an on-site loop. Sites can only be connected with paths if they are coupled via terms in the Hamiltonian.
- The relative sign of a diagram is $(-1)^{p+1}$ where p is the total number of loops encircling more than one site.

Each diagram is a term in $det_{1N}(EI - H)$. Each path connecting different sites contributes with its coupling element in the Hamiltonian. Each on-site loop contributes a factor $(E - \epsilon)$ where ϵ is the onsite energy of the site. For the remainder of the text we set the Fermi energy to zero and treat only the π-system with a p_z orbitals on each atom.

Supplementary Note 5 | Markussen diagrams for the molecules:

For the **dha** molecules, the π-system of the cyano groups are decoupled from the rest of the π-system. This means that both $det_{1N}(EI - H\ )$ and $\det(EI - H - \Sigma_L - \Sigma_R)$ share a common set of disconnected diagrams that cancel and therefore do not contribute to the transmission. For the **vhf**

molecules, the cyano groups are coupled directly to the tunneling path and therefore contribute to transport.

Details are presented in Supplementary Figure 17-19.

Supplementary Note 6 | Relating Markussen diagrams to DFT.

The transmission was calculated using parameters from GPAW and a modified version of the transport code supplied with ASE. The protonated molecules were relaxed in the gas phase to within a maximum force of 0.04 eV/Å using the PBE functional and a dzp basis set. The thiol groups were deprotonated and the molecule was positioned such that the S atoms sat 2.3 Å above two fcc111 Au in a hollow site position as shown in paper. The Hamiltonian was constructed using a 4x4 Monckhorst-Pack k-point sampling from which the transmission was calculated. The difference in conductance between **dha-*6*** and **dha-*7*** is more than an order of magnitude due to the destructive interference occurring in the middle of the band-gap. The calculated transmissions of the **vhf**s show a clear interference feature shifted away from the middle of the gap. The transmission peaks of **dha-*7*** and the **vhf**s do not go to one. We ascribe this to the bent character of the molecule of which leads to more localized orbitals that do not couple equally well to both orbitals and the difference in coupling to the electrodes since the geometry of the junction is not relaxed. This should not be important for off-resonant transport. For a π-system bent perpendicular to its plane we should expect a decrease in conductance roughly proportional to $\cos^2(\theta)$ where θ is the bending angle. For a bend of 45 ° this should constitute a decrease in conductance of a factor of ~0.5. The experimentally observed difference in conductance is much larger than this. Therefore, the difference in conductance must primarily be an effect of the electronic structure such as a larger band-gap of **vhf** and possibly some effect of the destructive interference. Similarly, the lower conductance of **dha-*7*** is most probably due to an electronic effect of which the destructive interference appears to be the most likely.
