# Supplementary material for: Single-molecule detection of dihydroazulene photo-thermal reaction using break junction technique
Source: Nat Commun. 2017 May 22;8:15436. doi: 10.1038/ncomms15436 (PMC5477511; doi:10.1038/ncomms15436)
Supplement: Peer review file [file ncomms15436-s2.pdf]

Reviewers' comments:

Reviewer #1 (Remarks to the Author):

Break junction measurements are regarded as a basic tool to determine the conductance of single-molecule nanowires. However, there are only very limited examples, where in-situ chemical reactions could be followed on the level of single molecules. The authors present a beautiful experimental study, where the temporal evolution of a photo-thermal reaction can be followed by the break junction data. The three possible molecular states (dha6, vhf, dha7) have well distinguishable molecular conductances, and the relative conductances are also understood by DFT transport calculations, highlighting the role of destructive interference in vhf and dha7. Under in situ UV irradiation measurements the single-molecule conductance data clearly demonstrate the saturating increase of the vhf content around the junction, whereas ex-situ heat treatment can reverse the process. The authors also demonstrate the different evolution of the thermal reaction around the gold surface, namely that the transformation of vhf to dha6 is preferred, whereas in solution environment the two end states (dha6 and dha7) have similar probability. In my view this clear temporal resolution of a photo-thermal reaction on the single-molecule level is a very important and pioneering result, so scientifically the paper could meet the requirements of Nature Communications. However, the quality of the presentation, especially the experimental figures does not fit to the high standard of Nature Publishing journals. My criticism mostly concerning this aspect is given bellow.

1.On lines 41-43 the authors write that they are not limited to measuring average characteristics of molecule ensembles, rather the reaction kinetics of single-molecule device is followed. This statement is a bit misleading, as the presented measurements are based on a huge statistical data of thousands of independent single-molecule nanowires, therefore not the reactions of a single molecule are followed. The advantage compared to macroscopic molecule ensembles is evident, but this statements should be phrased more precisely.

2.In Fig1b the scale of the histograms is missing (counts).

3.On the 2D conductance-displacement histograms the blue color scale is not a good choice, there are several better visualizations of such plots in the literature. Furthermore the scale bar only presents the two limits, so it is not clear, whether linear scaling is applied or not. The quality of these figures could be improved.

4.The 2D correlation histogram is strange, since -according to Ref. 21- it should exhibit perfect correlation at the diagonal. Why is it not satisfied on the figure? The color scale is also strange with the sharp change from yellow to red at 1, and the broad green part around zero.

5.Fig.2c is not well presented. The area graphs show at least 5 different colors, it is hard to follow which color corresponds to which histogram (the same holds for Fig.3c). Also the black/blue lines are hardly distinguishable. The colors of the related blue/black dotted rectangles in Fig2b are also hardly distinguishable on a printed page. A better way of presentation should be chosen, probably 2 out of 3 histograms should be only a line graph.

6.In Fig.2d the vertical scales are missing, it is not clear, whether all panels use the same scale or not.

7.The exponential fitting in Fig.2b is strange, the blue line should follow the formula P=P_0+(1-P0)*exp(-R_0*t), where P_0 is not the initial value of the percentage (as written on line 129), but the final value. The red line is even more disturbing, as this exponential fit should saturate at P=1, i.e. it should be a fitting curve like P=1-(1-P_0)*exp(-R_0*t). The red fitting curve is physically nonsense, as it would give P>1 at later times. The authors should correct this, and should comment, why the experimental data are different from an exponential saturation along the heating process.

8.The fine details of the ex-situ heating process are not given in the manuscript, it would be important to include these in the supporting information.

9.The meaning of the different symbols in Fig.3a and Fig.3d are not clarified in the caption in proper detail.

10.It would be useful to show the control experiment in the supporting information, where a similar measurement is done in the same solvent, but without adding the molecules, this would be a good reference to demonstrate the cleanliness of the measurement setup.

11.The discussions imply, that the photo-thermal reaction differs at the gold surface and in the solution. As the most prominent difference, the vhf is always transformed to dha6 at the surface, whereas in the solution both dh6 and dha7 appear as an end product of the thermal reaction. As the measurements are performed in a liquid cell, further away from the sample everything should work normally, like in a solution environment. I can imagine several possibilities: (i) the thermal reaction is catalyzed by the surface, and in the solution the temperature is not high enough to induce this reaction. (ii) there is an inequilibrium around the surface, where only dha6 is produced, whereas further away in the solution one would also find dha7 after the ex-situ heating. (iii) all the molecules are sitting on the surface, and so only the surface reaction is relevant. The different scenarios could be probably checked experimentally. In case (i) a higher temperature treatment would yield dha7 in the solution, which could be detected by the conductance histogram. In case (ii) a control measurement in the same solution, using a new sample should yield dha7 in the histogram. The authors should comment these issues.

Reviewer #2 (Remarks to the Author):

This manuscript discusses aspects of molecular switching in a system of dihydroazulene (dha) and/or vinylheptafulvene (vhf). Beginning initially with some isomer of dha, UV light transforms the system to vhf, where heat converts the molecule back to dha. Some aspects of this work are very similar to previous studies of photo-induced molecular switching, but there is one particularly novel aspect here that makes it potentially interesting for readers of Nature Communications. The authors consider two isomers of dha for the initial state. In solution, the back-reaction from vhf to dha produces both isomers, whereas the back-reaction in a molecular junction exclusively prefers one isomer. That a molecular junction could be used to study specific chemical reactions in such a manner is an intriguing idea that should be published in Nature Communications. However, it sometimes seemed that the authors rushed to submit this manuscript. A thorough proofreading should be performed, and the authors should also address the following points, before this paper can be accepted.

1) There seem to be too many main points to this communication. Some are of interest; for example, that the stoichiometry of the back-reaction is different in solution vs. in a molecular junction. However, others (photo-induced molecular switches, destructive interference features in some molecules but not others) have been rehashed in numerous previous studies. I think refocusing this paper to emphasize the novel points will make it more noteworthy. Elaborations of this point will be presented below.

2) In schematic 1, I believe the one-way arrow from vhf to dha-7 should go in the other direction, and be labelled “UV”. The figure, as is, seems completely contrary to the results discussed in the text. The authors may want to keep the current “heating” arrow, but put it in a different color to denote “in solution only”.

3) The paragraph beginning on line 110 is very unclear. Line 113 mentions a “relative plateau length”. What is this, exactly? How is it determined? The threshold {delta z} = 1 nm also seems arbitrary. Can the authors please provide more detail on justifying it? Do the results change if {delta z} changes significantly? How does {delta z} = 1 nm compare to the molecular length? The use of the word “setting” in line 118 seems weird. I’m under the impression that {delta z} is a property of a conductance trace, and isn’t something the authors can “set”. Perhaps they meant “filtering” or the like? I’m not sure what to make of the sentence beginning with “It is also found ..." on line 121. Why do the authors fit the {delta z} histograms with Gaussians to determine the ratio? Couldn’t they just count the number of traces that fall into either category and take the ratio directly?

4) Lines 134, 138, and 144 refer to the “reversible reaction”, meaning the back-reaction from vhf to dha-6. The authors have not yet discussed the irreversible reaction from dha-7 to vhf, making this nomenclature a bit odd. Using “back-reaction” instead of “reversible reaction” makes it clearer, at least to me.

5) Line 145 refers the reader to the supporting information for more details on why the dha-6 to vhf reaction does not run to “completion” in a molecular junction. The supporting information is completely unstructured, and it is hard to locate this discussion within it.

6) The authors assume, in lines 202-203, that the various vhf isomers are in equilibrium at all

times during the experiment because the isomers all have roughly the same energies. Is this justification valid? Thermodynamically, it's fine, but the energies alone don't tell us about the kinetics.

7) Building on point 1, the paragraph beginning on line 214 can be greatly condensed or even removed. None of the analysis here is new, nor does the analysis significantly help us understand the chemical physics of this system. For instance, the destructive interference features may explain why dha-7 is less conductive than vhf, which is less conductive than dha-6, but is that really relevant to the main message? A sentence, two at the most, is probably sufficient. Figure 7 can similarly be condensed to only panel a.

8) I think a few additional calculations, similar to those performed for Figure 7a, would be extremely beneficial. Could the authors put small clusters of gold (or some other metal) atoms around the molecules, at the appropriate binding sites, to estimate how the junction changes the energetics from the solution phase? These numbers would remove quite a bit of the speculation offered by the authors. Their hypotheses are reasonable, but these results would help support (or refute) them.

For other minor points:
9) The red dotted lines to denote the regions in Figure 1d are hard to read, especially given the amount of red areas in the correlation histogram. Making the lines black would add more contrast and guide the reader's attention to the important aspects.

10) Figure 2's caption had quite a few typos or unclear passages. The typos I found are all in line 97, "Inverted" should not have a capital "I", there should be "is" in "and fitted", and "red cure" should be "red curve". Line 100 references a "blue dash[ed] box", but there are two of them in panel b. The start of the next sentence, "Due to all of data is recorded during ..." is unclear. Furthermore, the caption does not adequately justify the presence of panel c. Such a discussion can be found in lines 150-152, but should be mirrored in the caption. Finally, in line 102, should "different reaction states" be "difference reaction times"?

11) In lines 108-109, approximately how many traces were neither dha or vhf, or were indeterminate?

12) It is stated in line 128 that the irradiation reaction reaches saturation after 80 minutes, but Figure 2b suggests the time is closer to 150 minutes.

13) In line 129, "hollow triangles" should be "upright triangles", as both symbols are hollow.

14) Is Figure 5 necessary?

Point-by-point response to the referees' comments

Reviewer #1 (Remarks to the Author):

Break junction measurements are regarded as a basic tool to determine the conductance of single-molecule nanowires. However, there are only very limited examples, where in-situ chemical reactions could be followed on the level of single molecules. The authors present a beautiful experimental study, where the temporal evolution of a photo-thermal reaction can be followed by the break junction data. The three possible molecular states (dha6, vhf, dha7) have well distinguishable molecular conductances, and the relative conductances are also understood by DFT transport calculations, highlighting the role of destructive interference in vhf and dha7. Under in situ UV irradiation measurements the single-molecule conductance data clearly demonstrate the saturating increase of the vhf content around the junction, whereas ex-situ heat treatment can reverse the process. The authors also demonstrate the different evolution of the thermal reaction around the gold surface, namely that the transformation of vhf to dha6 is preferred, whereas in solution environment the two end states (dha6 and dha7) have similar probability. In my view this clear temporal resolution of a photo-thermal reaction on the single-molecule level is a very important and pioneering result, so scientifically the paper could meet the requirements of Nature Communications. However, the quality of the presentation, especially the experimental figures does not fit to the high standard of Nature Publishing journals. My criticism mostly concerning this aspect is given bellow.

1. On lines 41-43 the authors write that they are not limited to measuring average characteristics of molecule ensembles, rather the reaction kinetics of single-molecule device is followed. This statement is a bit misleading, as the presented measurements are based on a huge statistical data of thousands of independent single-molecule nanowires, therefore not the reactions of a single molecule are followed. The advantage compared to macroscopic molecule ensembles is evident, but this statements should be phrased more precisely.

Response:

Thanks for pointing out. To avoid the confusion, we changed the sentence to be "More importantly, break-junction techniques could provide a means to statistically quantify the photo-reaction kinetics of single-molecule device connected between two electrodes, which

may offer some new understanding beyond measuring an ensemble of molecules as in other spectroscopies."

2.In Fig1b the scale of the histograms is missing (counts).

Response:

The scale is added, and the figure is replaced.

3.On the 2D conductance-displacement histograms the blue color scale is not a good choice, there are several better visualizations of such plots in the literature. Furthermore, the scale bar only presents the two limits, so it is not clear, whether linear scaling is applied or not. The quality of these figures could be improved.

Response:

We add the label in the scale color bar and also in caption to state the linear scaling is applied. Concerning color scale selected for visualization, we prefer to keep the current color map to be consistent with our previous papers.

4.The 2D correlation histogram is strange, since -according to Ref. 21- it should exhibit perfect correlation at the diagonal. Why is it not satisfied on the figure? The color scale is also strange with the sharp change from yellow to red at 1, and the broad green part around zero.

Response:

In the new figure 1d, we replaced the color scale for better contrast. Thus perfect correlation could be demonstrated at the diagonal as shown in the black line with blue color. We appreciate the reviewer for the nice suggestions.

5.Fig.2c is not well presented. The area graphs show at least 5 different colors, it is hard to follow which color corresponds to which histogram (the same holds for Fig.3c). Also the black/blue lines are hardly distinguishable. The colors of the related blue/black dotted rectangles in Fig2b are also hardly distinguishable on a printed page. A better way of presentation should be chosen, probably 2 out of 3 histograms should be only a line graph.

Response:

Thanks for your suggestions. The conductance histogram is shown as the simple curves with three different colors representing three compounds. The figures are replaced. In Fig2b, to avoid the confusing of the colors, the rectangles are shown in solid lines with different colors for better desciption.

6.In Fig.2d the vertical scales are missing, it is not clear, whether all panels use the same scale or not.

Response:

The scale is added, and all panels are using the same scale.

7.The exponential fitting in Fig.2b is strange, the blue line should follow the formula P=P_0+(1-P0)*exp(-R_0*t), where P_0 is not the initial value of the percentage (as written on line 129), but the final value. The red line is even more disturbing, as this exponential fit should saturate at P=1, i.e. it should be a fitting curve like P=1-(1-P_0)*exp(-R_0*t). The red fitting curve is physically nonsense, as it would give P>1 at later times. The authors should correct this, and should comment, why the experimental data are different from an exponential saturation along the heating process.

Response:

Thanks for your correction, and we have corrected that P0 to be final value for blue line.
Concerning the red part, our idea is to make the decay constant comparable with previous literature that we applied the same fitting, P=P0+A×exp (R0t) as in Ref 23 (*Nano Lett.* 2013, 13, 337−343) where the P0 is the initial value. After the fitting, the P0=0.25, which is in agreement with the experiment data.
We agree that P=1-(1-P0)*exp(-R0*t) could be more proper to describe the reaction in solution, and we have also tried to fit the data (thermal process) by the P=1-(1-P0)*exp(-R0*t). However, it seems the equation doesn't fit the experimental data well (as shown below). The disagreement suggest that **vhf** is not decaying to **dha** with a first-order decay in a junction, which may be due to the fact that constraints of the junction alter the kinetics in some way.

To clarify this point, we add the discussion in the main text "It is also found that the kinetics of the back reaction is different from the exponential saturation, which suggest the constraints of the junction may alter the reaction kinetics."

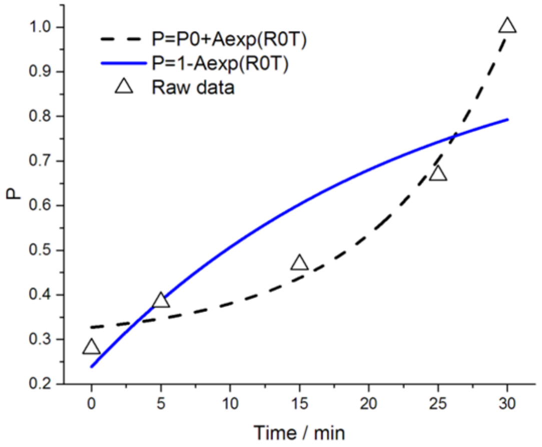

8.The fine details of the ex-situ heating process are not given in the manuscript; it would be important to include these in the supporting information.

Response:

We added the description of ex-situ heating process into methods.

"In the ex-situ heating process, the solution was put in a water bath with constant temperature (65 ^{o}C). After set waiting time (0 min, 5 min, 15 min, 25 min and 30 min), we took 200 μL solution into the liquid cell of MCBJ for conductance measurements."

9.The meaning of the different symbols in Fig.3a and Fig.3d are not clarified in the caption in proper detail.

Response:

We modify the figure caption by adding more descriptions.

Figure 3 | Photo/thermal conversion of the isomer by MCBJ. **a** Plot of reversible conversion of **dha-*6*↔vhf** in 6 cycles. The solid circle means the conductance, related to left scale, while the hollow circle is the percentage of **dha-*6*,** related to right scale**.** **b** Schematic for the conversion of **dha-*7*** with the treatments by UV irradiation and heating. **c** One-dimensional (1D) conductance histogram from different states. The black is from the initial **dha-*7*** state, the red is for the state after UV irradiation to **vhf**, and the blue is from the

conductance measurements for solution after heating process. **d** Plot of conductance switch cycles starting from **dha-7**. The error bars of the conductance are determined from the conductance analysis based on Gaussian Function (s.d.). The solid circle means the conductance, related to left scale, while the hollow circle is the percentage of **dha-6,** related to right scale**.**

10.It would be useful to show the control experiment in the supporting information, where a similar measurement is done in the same solvent, but without adding the molecules, this would be a good reference to demonstrate the cleanliness of the measurement setup.

Response:

We add the experiment in supplementary information as Supplementary Figure 21 and also add the reference in the method section.

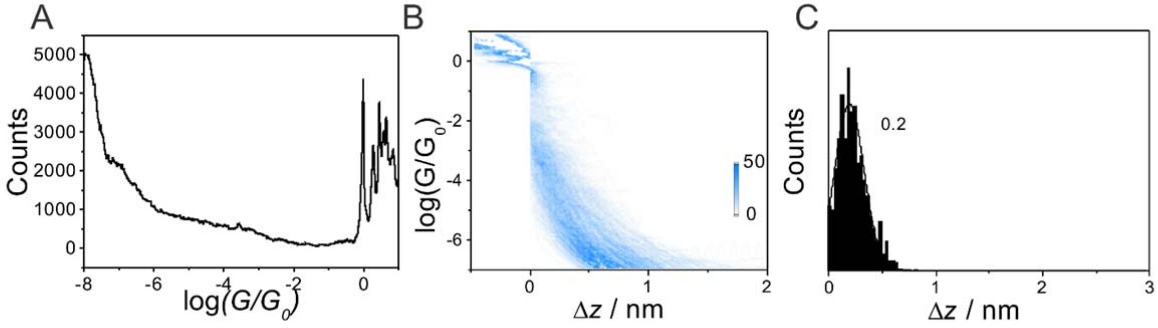

Supplementary Figure 21 | MCBJ experiment in solvent without molecules. The conductance histogram in the pure solvent without adding molecules. A: 1D conductance histogram; B: 2D conductance histogram C: the relative displacement distribution histogram determined from the conductance range between 0.7 G_0 to 10^{-3} G_0. Clean background suggested no molecular junction are formed during the experiment with pure solvent.

11.The discussions imply, that the photo-thermal reaction differs at the gold surface and in the solution. As the most prominent difference, the vhf is always transformed to dha6 at the surface, whereas in the solution both dh6 and dha7 appear as an end product of the thermal reaction. As the measurements are performed in a liquid cell, further away from the sample everything should work normally, like in a solution environment. I can imagine several possibilities: (i) the thermal reaction is catalyzed by the surface, and in the solution the temperature is not high enough to induce this reaction. (ii) there is an inequilibrium around the surface, where only dha6 is produced, whereas further away in the solution one would

also find dha7 after the ex-situ heating. (iii) all the molecules are sitting on the surface, and so only the surface reaction is relevant. The different scenarios could be probably checked experimentally. In case (i) a higher temperature treatment would yield
dha7 in the solution, which could be detected by the conductance histogram. In case (ii) a control measurement in the same solution, using a new sample should yield dha7 in the histogram. The authors should comment these issues.

Response:

Thanks for your suggestions.

We use new MCBJ sample sheets to carried out the measurement of the solution after UV irradiation and thermal treatment. Supplementary Figure 24A shows the conductance properties of solution with UV irradiation for 15 mins measured by new sample, and Supplementary Figure 24B shows conductance of solution with thermal treatment (50 min) for the **vhf** solution. We got only one conductance feature after the thermal treatment using a new MCBJ sample sheet, which could be assigned to ***dha-6***. No signal of ***dha-7*** (located at 10^{-6} G_0) was found in the control experiment. While in the solution, after thermal treatment of vhf solution, we got the mixture of ***dha-6*** and ***dha-7*** in UV-Vis absorption spectra, as shown in Supplementary Figure 9. The difference suggested dha-6 are highly preferred in a junction configuration.

We added the control experiments in supporting information as Supplementary Figure 24A.

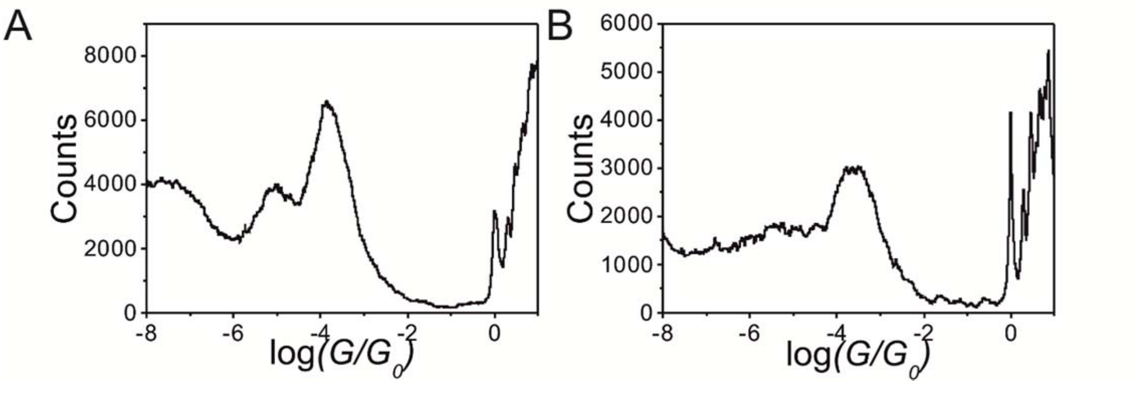

"Supplementary Figure 24 | Control experiments using new MCBJ samples to compare reaction in junction and in solution A: the conductance histogram of solution (***dha-6***) with UV irradiation (15 min) measured by a new MCBJ sample; B: the conductance histogram of solution (**vhf**) after thermal treatment (50 min) measured by a new MCBJ sample. We used a new MCBJ sample sheet to carry out the measurement of the solution after UV irradiation

and thermal treatment as control measurement. We got only one conductance feature after the thermal treatment using a new MCBJ sample sheet, which could be assigned to **dha-6**. No signal of **dha-*7*** (located at 10^{-6} G_0) was found in the control experiment. While in the solution, after thermal treatment of **vhf** solution, we got the mixture of **dha-*6*** and **dha-*7*** in UV-Vis absorption spectra, as shown in Supplementary Figure 9. The difference suggested **dha-*6*** are highly preferred in a junction configuration."

To further understand the difference, we have added the energy calculations for the dha and vhf isomers when placed in a junction between the two gold electrodes. We find that **dha-*6*** is more stable than **dha-*7*** by 3.01 kcal mol^{-1} in the junction, while the difference was only found to be 0.39 kcal mol^{-1} for the molecules in solution. Thus, we see that the junction significantly alters the relative energies that the molecular junction prefers the conversion to **dha-*6***. A new paragraph is included in the manuscript.

"For comparing the energies of the two molecules **dha-*6*** and **dha-*7*** when placed in a junction (ensuring interactions between molecule and metal leads), we performed combined QM/MM calculations[26] using the program Dalton QCP.[27] The gold electrodes are built as two hemispheres from a fcc unit cell and described using molecular mechanics. Each gold cluster consists of 262 Au atoms, where each gold atom is assigned a polarizability of 31.04 au. The molecule faces the [111] surface with the axis through the two sulfur atoms oriented perpendicular to the gold surface. The DFT geometry-optimized molecules were inserted between the two gold clusters and then single point energy calculations were performed (CAM-B3LYP//cc-pVDZ). Interestingly, we find that **dha-*6*** is more stable than **dha-*7*** by 3.04 kcal mol-1 in the junction, while the difference was only 0.39 kcal mol-1 for the molecules in solution (acetonitrile). A smaller difference between the reactive s-cis ***Z*-vhf** and s-cis ***E*-vhf** isomers was obtained, with the ***Z*-vhf** being 0.72 kcal mol^{-1} more stable than the ***E*-vhf**. Although the slightly more energetic ***E*-vhf** is the precursor for **dha-*6***, it pays off overall from a thermodynamic point of view to form **dha-*6*** in the junction. We were not able to perform reliable TS calculations in the junction in order to ascertain whether the kinetics of ring closure also agrees with experiment. Nevertheless, we have previously observed that the larger the **dha-vhf** energy difference, the faster is the **vhf**-to-**dha** conversion.[27,28]"

Additional material has been added to the Supplementary Table 1 and Fig. 20.

"

	**Solvent**	**Relative**	**Junction**	**Relative**

	(kcal/mol)	**Energies** (kcal/mol)	(kcal/mol)	**Energies** (kcal/mol)
**dha-*6***	-1292703.47	0.00	-1292070.76	0.00
**TS6**	-1292673.89	29.58	-	-
***E*-vhf** (s-*cis*)	-1292698.29	5.18	-1292057.52	13.24
***Z*-vhf** (s-*cis*)	-1292698.32	5.15	-1292058.24	12.52
**TS7**	-1292674.59	28.88	-	-
**dha-*7***	-1292703.08	0.39	-1292067.73	3.04
***E*-vhf** (s-*trans*)	-1292700.21	3.26	-1292061.43	9.33
***Z*-vhf** (s-*trans*)	-1292700.35	3.12	-1292065.47	5.29
**Rot**	-1292698.28	5.19	-	-

Supplementary Table 1 | Energies for molecules in solvent (acetonitrile) and junction. For the calculations in solution, the molecules are end-capped with SH groups (not SAc), while they are end-capped with S in the junction. **TS6** is the transition state structure from ***E*-vhf** (s-*cis*) to **dha-*6***; **TS7** is the transition state structure from ***Z*-vhf** (s-*cis*) to **dha-*7***; **Rot** is the TS structure between *Z*- and *E*-**vhf** in s-*cis* conformations.

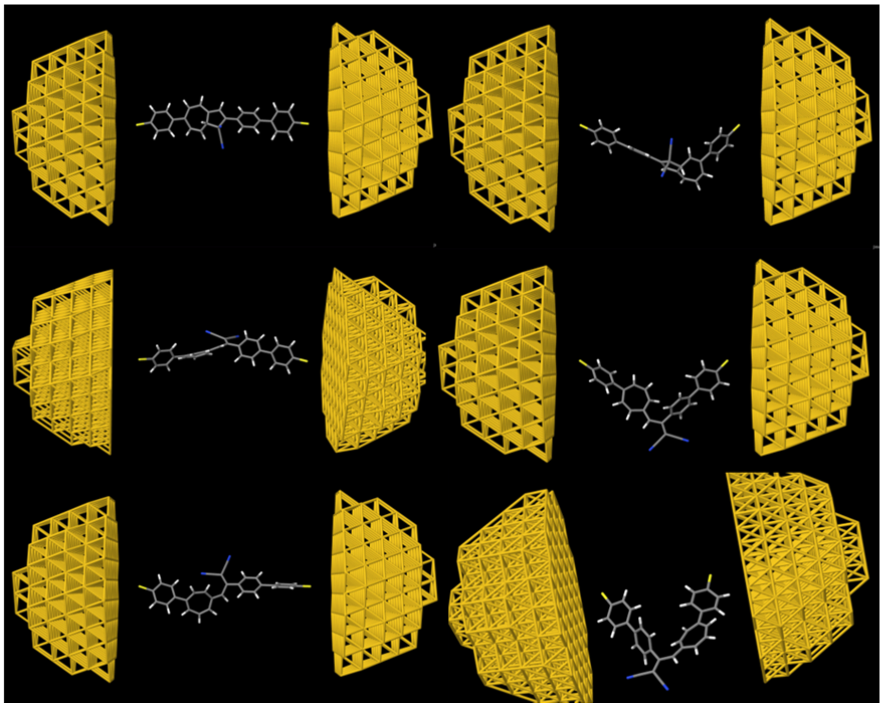

Supplementary Figure 20 | Schematic representations of the junction set-up. From top to bottom (left / right): **dha-*6* / dha-*7***, ***E*-vhf** (s-*cis* / s-*trans*), ***Z*-vhf** (s-*cis* / s-*trans*). A combined QM/MM method was used to calculate the energy of **dha-*6*** and **dha-*7*** in a junction and their **vhf** forms, ensuring the inclusion of the screening effect of the metal leads. The molecule is treated quantum mechanically by DFT using the long-range corrected functional CAM-B3LYP and the correlation consistent basis set cc-pVDZ. The molecular mechanically gold electrodes are built as two hemispheres from a fcc unit cell. Each gold cluster is consisting of 262 Au atoms, where each gold atom is assigned with a polarizability of 31.04 au. The molecule faces the [111] surface with the axis through the two sulfur atoms standing perpendicular to the gold surface."

Reviewer #2 (Remarks to the Author):

This manuscript discusses aspects of molecular switching in a system of dihydroazulene (dha) and/or vinylheptafulvene (vhf). Beginning initially with some isomer of dha, UV light transforms the system to vhf, where heat converts the molecule back to dha. Some aspects of this work are very similar to previous studies of photo-induced molecular switching, but there is one particularly novel aspect here that makes it potentially interesting for readers of Nature Communications. The authors consider two isomers of dha for the initial state. In solution, the back-reaction from vhf to dha produces both isomers, whereas the back-reaction in a molecular junction exclusively prefers one isomer. That a molecular junction could be used to study specific chemical reactions in such a manner is an intriguing idea that should be published in Nature Communications. However, it sometimes seemed that the authors rushed to submit this manuscript. A thorough proofreading should be performed, and the authors should also address the following points, before this paper can be accepted.

1) There seem to be too many main points to this communication. Some are of interest; for example, that the stoichiometry of the back-reaction is different in solution vs. in a molecular junction. However, others (photo-induced molecular switches, destructive interference features in some molecules but not others) have been rehashed in numerous previous studies. I think refocusing this paper to emphasize the novel points will make it more noteworthy. Elaborations of this point will be presented below.

<u>Response:</u>

Thanks for your suggestions. We agree that the stoichiometry of the back-reaction is maybe the most important result, and we also believe that the other points are actually contributing to a coherent story. While significant work has focused on the dithienylethene photoswitching, the consequences of cross-conjugation and deviations from planarity in regard to conductance properties have never been elucidated for the **dha-vhf** switch in the coherent transport regime. And in our work, it is particularly interesting that we have 3 isomers (**dha-6**, ***E/Z*-vhf** and **dha-7**), and not only 2, where both planarity and conjugation are changing. The overall results on experimental/theoretical conductance properties, isomer geometries (calculated and X-ray), destructive interference patterns, and optical properties (**dha-6** *vs* **dha-7**) seem to converge nicely.

To further strengthen the connection among these points and the coherence of the story, we revised the description in abstract to

"Here, we explore the potential of single-molecule break junction technique in the detection of photo-thermal reaction processes of a photochromic dihydroazulene / vinylheptafulvene system. Statistical analysis of the break junction experiments provides a quantitative approach for probing the reaction kinetics and reversibility, including the occurrence of isomerization during the reaction."

We also reorganized the last paragraph of introduction to be

"We employ the Mechanically Controllable Break Junction (MCBJ) technique to detect the photo-thermal reaction processes of a tailor-made photochromic dha / vhf system. Three distinguishable conductance states were observed experimentally, while reversible and distinct changing of the single molecular conductance was observed between two of the states. Benefiting from the well-distinguished conductance states, the reaction kinetics and reversibility could be studied via statistical analysis of the conductance-distance traces, and it is found that the reaction in the junction did not follow those observed in solution, which agrees well with the energy calculation. Density functional transport calculations reproduced the relative conductance of these molecular states and identified a changing destructive interference effect as being responsible for the large span in conductance."

And reorganize the first paragraph of discussion part to be:

"To conclude, we have developed an approach to detect the photo-thermal reaction of dha/vhf system using single-molecule break junction technique. We have demonstrated that the single-molecule junction technique is able to track the photo-thermal reaction, and to evaluate the reversibility and multiple switching possibilities during the reaction using the well-distinguished conductance states of single-molecule dha/vhf junctions. The combined

transport calculations suggest that these distinct conductance states were ascribed to the introduction and shifting of interference features in the molecular transmission."

2) In schematic 1, I believe the one-way arrow from vhf to dha-7 should go in the other direction, and be labelled "UV". The figure, as is, seems completely contrary to the results discussed in the text. The authors may want to keep the current "heating" arrow, but put it in a different color to denote "in solution only".

Response:

The schematic has been changed, and we add "and the conversions observed in junction" in the caption.

3) The paragraph beginning on line 110 is very unclear. Line 113 mentions a "relative plateau length". What is this, exactly? How is it determined? The threshold {delta z} = 1 nm also seems arbitrary. Can the authors please provide more detail on justifying it? Do the results change if {delta z} changes significantly? How does {delta z} = 1 nm compare to the molecular length? The use of the word "setting" in line 118 seems weird. I'm under the impression that {delta z} is a property of a conductance trace, and isn't something the authors can "set". Perhaps they meant "filtering" or the like? I'm not sure what to make of the sentence beginning with "It is also found ..." on line 121. Why do the authors fit the {delta z} histograms with Gaussians to determine the ratio? Couldn't they just count the number of traces that fall into either category and take the ratio directly?

Response:

Sorry for the confusion. The relative plateau length is the value of the stretching distance of Au electrodes, which is determined from the stretched distance of the two electrodes when the conductance changes within certain range ($10^{-4.5}$ ~10^{-6} G_0 for **vhf**), $10^{-3.0}$ ~ $10^{-4.5}$ G_0 for **dha-6** as indicated in the caption of figure 2). It is defined as the "relative" due to the missing of the "snap-back" distance after the breaking of Au-Au atomic contact. (reference 20). To be consistent with our previous paper, we change the relative plateau length to relative displacement and add the reference.

As shown in Figure 2a, after Gaussian fitting, two significant peaks were shown, marked as T and M. T is assigned to the pure tunnelling through solvent without the formation of

molecular junction, and M is assigned to molecular junction. In this case, value of 1 nm comes from the cross points of the two fitted peak, and it varied from different histograms with different conditions in the range of 0.7-1.2 nm. The values of the **dha** percentage are determined from the area ratio of two peaks using Gaussian fitting, which are independent from the cross point of the two fitted peaks.

For better description, we reorganize the paragraph to be:

"Figure 2a shows the typical relative displacement distributions of conductance from MCBJ measurements with *in-situ* UV irradiation (30 min). To determine the time dependent junction evolution, 500 conductance curves are used for the statistical analysis for each time period. Using the conductance range of $10^{-3.0}$ ~ $10^{-4.5}$ G_0 (region 2) and $10^{-4.5}$ ~10^{-6} G_0 (region 1), we are able to construct the relative displacement distribution for **dha** and **vhf**, respectively. In the relative displacement distributions shown in Figure 2a,[20] the Gaussian fitting of the two peak distributions suggested that the traces with molecular junction (marked with M) are typically longer than 1 nm, while the tunneling traces without molecular plateau are typically at around 0.5 nm (marked with T). In this way, we could determine the peak area ratio of **dha-*6*** or **vhf** junctions from the Gaussian fitting of displacement distribution of all conductance traces including the **dha-*6*** (or **vhf**) junction traces and tunneling traces (see supplementary Fig. 22 for more details). Therefore, the percentage of **dha-*6*** junctions in all molecular junctions could be determined for the further kinetics analysis."

We have also added the detailed description of peak area ratio determination and validation of the approach as supplementary Fig. 22.

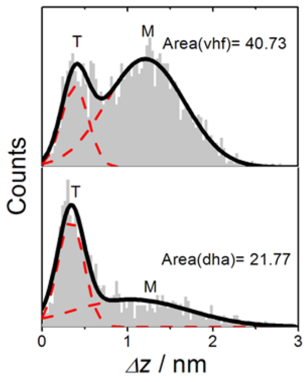

"Supplementary Figure 22 | Determination of the percentage of dha-*6* junction from relative displacement distributions. Relative displacement distributions of MCBJ

conductance measurements with the *in-situ* UV irradiation (120 min). The conductance regions of the top and below panel are $10^{-4.5}$ ~10^{-6} G_0 (**vhf**), $10^{-3.0}$ ~ $10^{-4.5}$ G_0 (**dha-*6***), respectively. The relative displacement distribution histogram was fitting by Gaussian function, and the peaks assigned to molecular feature are marked with M. The peak areas were integrated to determine the number of traces with specific molecular feature. Thus we could determine area ratio of **dha-*6*** is 0.34 (= 21.77/(21.77+40.73)), which is relevant to the percentage of **dha-*6*** molecules in this experimental condition. To further validate the determination of area ratio in another way, we could also count the number of traces with relative displacement longer than 0.72 nm, where 0.72 nm was determined from the cross point of the two peak fitting. We find that the number of trace for vhf is 108, and the number for **dha-*6*** is 63. the percentage of **dha-*6*** is around 0.33 (=63/(63+108)), which agree with the area ratio method we applied in this work well."

4) Lines 134, 138, and 144 refer to the "reversible reaction", meaning the back-reaction from vhf to dha-6. The authors have not yet discussed the irreversible reaction from dha-7 to vhf, making this nomenclature a bit odd. Using "back-reaction" instead of "reversible reaction" makes it clearer, at least to me.

Response:

We agree to use the "back reaction" instead of "reversible reaction".

5) Line 145 refers the reader to the supporting information for more details on why the dha-6 to vhf reaction does not run to "completion" in a molecular junction. The supporting information is completely unstructured, and it is hard to locate this discussion within it.

Response:

Sorry for the confusion. We reorganize the supporting information. and modify the sentence to "It is also confirmed by the experiments with longer time of UV-irradiation (Supplementary Figure 23)."

6) The authors assume, in lines 202-203, that the various vhf isomers are in equilibrium at all times during the experiment because the isomers all have roughly the same energies. Is this justification valid? Thermodynamically, it's fine, but the energies alone don't tell us about the kinetics.

Response:

We have repeated the energy calculations for the dha and vhf isomers when placed in a junction. We find that **dha-*6*** is more stable than **dha-*7*** by 3.01 kcal mol^{-1} in the junction, while the difference was only found to be 0.39 kcal mol^{-1} for the molecules in solution (acetonitrile). Thus, we see that the junction significantly alters the relative energies. A smaller difference was obtained between the s-*cis* vhf isomers. A paragraph on these calculations has been added to the manuscript. We were unfortunately not able to achieve reliable values for the transition state structures (this is not even trivial to do in vacuum). We can therefore only make conclusions based on thermodynamics data and not kinetics data. But it is reasonable to assume that the most stable dha (**dha-*6***) is also formed faster than the less stable one. We have consistently observed this for other **vhf** to **dha** conversions – the larger the **vhf-dha** energy difference, the faster is the **vhf** to **dha** conversion (Cacciarini, M. *et al. Chem. Eur. J.* **2015**, *21*, 7454-7461; Skov, A. B. *et al. ChemPhotoChem*, In press. DOI: 10.1002/cptc.201600046).

7) Building on point 1, the paragraph beginning on line 214 can be greatly condensed or even removed. None of the analysis here is new, nor does the analysis significantly help us understand the chemical physics of this system. For instance, the destructive interference features may explain why dha-7 is less conductive than vhf, which is less conductive than dha-6, but is that really relevant to the main message? A sentence, two at the most, is probably sufficient. Figure 7 can similarly be condensed to only panel a.

Response:

Thanks for suggestions.

The stoichiometry studied in this work is based on the charge transport through molecular junctions, thus the understanding of charge transport becomes essential for the understanding of data. Actually at the beginning of this work we are not expecting that **dha-7** has orders of magnitude lower conductance than **dha-6** although their structures are quite similar. We think the discussion of charge transport through these derivatives will help the understanding of the single-molecule conductance experiments.

We condense the paragraph to be:

“Despite the chemical similarities of **dha-*6***, **vhf** and **dha-*7***, their transport properties are vastly different and followed the trend **dha-*6***>**vhf**> **dha-*7*** in the experiments. A theoretical

analysis using a diagrammatic approach first used by Markussen *et al.*[30, 31] combined with DFT transport caculations shows that **dha-*6*** has the highest conductance because it is both almost planar and does not show destructive interference (Figure 4b). Conversely, **dha-*7*** and **vhf** are bent and show destructive interference. The difference between these two systems results from the different energies at which the interference feature occurs. The diagrammatic description predicts no destructive interference for **dha-*6*** because it is possible to pair up all the non-traversed π-orbitals along the path connecting the electrodes for these molecules. However, this is not possible for **dha-*7*** and **vhf** as illustrated in Figure 4c. In **dha-*7***, the anchoring group is placed at C7, which causes the significant deviation of the π-system from planarity as also confirmed by X-ray crystallographic analysis (Figure 5), moreover, the nitrogen atoms are electronically decoupled from the rest of the π-system by saturated bonds and destructive interference therefore occurs in the middle of the band gap. This decoupling is not present in **vhf** and the anti-resonance is therefore shifted away from the middle of the band gap due to the electron-accepting character of CN. The calculated features of **dha-*6*** and **dha-*7*** are in line with their different optical properties determined experimentally (*vide supra*): **dha-*6*** exhibits a red-shifted longest-wavelength absorption maximum relative to **dha-*7***."

8) I think a few additional calculations, similar to those performed for Figure 7a, would be extremely beneficial. Could the authors put small clusters of gold (or some other metal) atoms around the molecules, at the appropriate binding sites, to estimate how the junction changes the energetics from the solution phase? These numbers would remove quite a bit of the speculation offered by the authors. Their hypotheses are reasonable, but these results would help support (or refute) them.

Response:

We agree and have now (as mentioned above) added relative energy calculations for the **dha** and **vhf** isomers in the junction. While the **dha** energies do not deviate significantly in solution, there is indeed a significant energy difference in the junction. The junction can thus strongly alter the energetics. We were not able to achieve reliable transition states in the junction, but we think the additional calculations still nicely support our hypothesis. At least thermodynamically, **dha-*6*** is the preferred isomer.
A new paragraph below is included in the manuscript.

“For comparing the energies of the two molecules **dha-*6*** and **dha-*7*** when placed in a junction (ensuring interactions between molecule and metal leads), we performed combined QM/MM calculations[26] using the program Dalton QCP.[27] The gold electrodes are built as two hemispheres from a fcc unit cell and described using molecular mechanics. Each gold cluster consists of 262 Au atoms, where each gold atom is assigned a polarizability of 31.04 au. The molecule faces the [111] surface with the axis through the two sulfur atoms oriented perpendicular to the gold surface. The DFT geometry-optimized molecules were inserted between the two gold clusters and then single point energy calculations were performed (CAM-B3LYP//cc-pVDZ). Interestingly, we find that **dha-*6*** is more stable than **dha-*7*** by 3.04 kcal mol-1 in the junction, while the difference was only 0.39 kcal mol-1 for the molecules in solution (acetonitrile). A smaller difference between the reactive s-cis ***Z*-vhf** and s-cis ***E*-vhf** isomers was obtained, with the ***Z*-vhf** being 0.72 kcal mol^{-1} more stable than the ***E*-vhf**. Although the slightly more energetic ***E*-vhf** is the precursor for **dha-*6***, it pays off overall from a thermodynamic point of view to form **dha-*6*** in the junction. We were not able to perform reliable TS calculations in the junction in order to ascertain whether the kinetics of ring closure also agrees with experiment. Nevertheless, we have previously observed that the larger the **dha-vhf** energy difference, the faster is the **vhf**-to-**dha** conversion.[27,28]”

Additional material has been added to the Supplementary Table 1 and Fig. 20.

“

	**Solvent** (kcal/mol)	**Relative Energies** (kcal/mol)	**Junction** (kcal/mol)	**Relative Energies** (kcal/mol)
**dha-*6***	-1292703.47	0.00	-1292070.76	0.00
**TS6**	-1292673.89	29.58	-	-
***E*-vhf** (s-*cis*)	-1292698.29	5.18	-1292057.52	13.24
***Z*-vhf** (s-*cis*)	-1292698.32	5.15	-1292058.24	12.52
**TS7**	-1292674.59	28.88	-	-
**dha-*7***	-1292703.08	0.39	-1292067.73	3.04
***E*-vhf** (s-*trans*)	-1292700.21	3.26	-1292061.43	9.33
***Z*-vhf** (s-*trans*)	-1292700.35	3.12	-1292065.47	5.29

**Rot**	-1292698.28	5.19	-	-

Supplementary Table 1 | Energies for molecules in solvent (acetonitrile) and junction. For the calculations in solution, the molecules are end-capped with SH groups (not SAc), while they are end-capped with S in the junction. **TS6** is the transition state structure from ***E*-vhf** (s-*cis*) to **dha-6**; **TS7** is the transition state structure from ***Z*-vhf** (s-*cis*) to **dha-*7***; **Rot** is the TS structure between *Z*- and *E*-**vhf** in s-*cis* conformations.

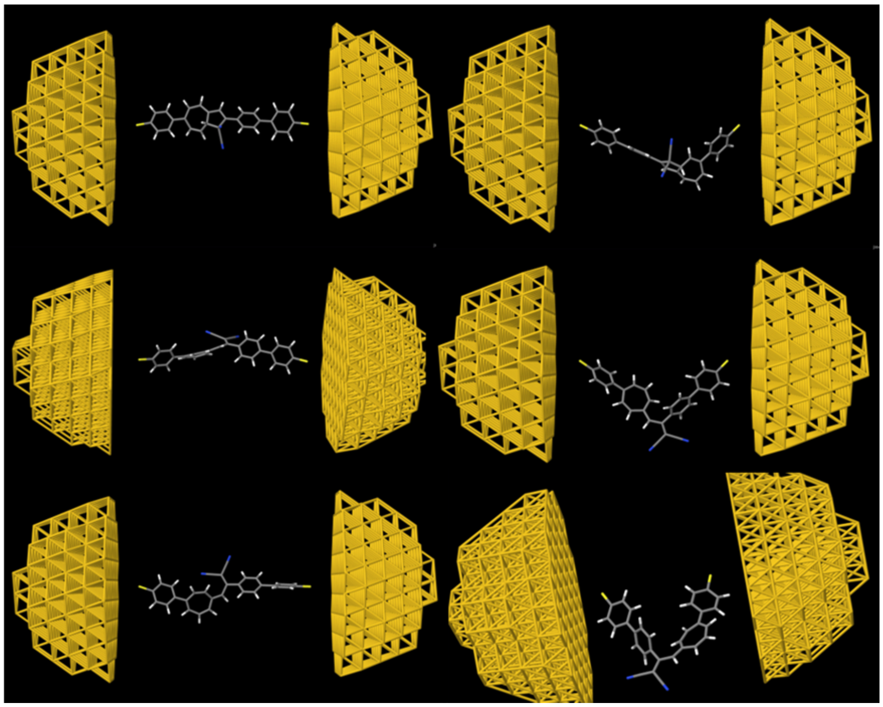

Supplementary Figure 20 | Schematic representations of the junction set-up. From top to bottom (left / right): **dha-*6*** / **dha-*7***, ***E*-vhf** (s-*cis* / s-*trans*), ***Z*-vhf** (s-*cis* / s-*trans*). A combined QM/MM method was used to calculate the energy of **dha-*6*** and **dha-*7*** in a junction and their **vhf** forms, ensuring the inclusion of the screening effect of the metal leads. The molecule is treated quantum mechanically by DFT using the long-range corrected functional CAM-B3LYP and the correlation consistent basis set cc-pVDZ. The molecular mechanically gold electrodes are built as two hemispheres from a fcc unit cell. Each gold cluster is consisting of 262 Au atoms, where each gold atom is assigned with a polarizability of 31.04 au. The molecule faces the [111] surface with the axis through the two sulfur atoms standing perpendicular to the gold surface."

For other minor points:

9) The red dotted lines to denote the regions in Figure 1d are hard to read, especially given the amount of red areas in the correlation histogram. Making the lines black would add more contrast and guide the reader's attention to the important aspects.

Response:

We redraw the figure to provide better contrast.

10) Figure 2's caption had quite a few typos or unclear passages. The typos I found are all in line 97, "Inverted" should not have a capital "I", there should be "is" in "and fitted", and "red cure" should be "red curve". Line 100 references a "blue dash[ed] box", but there are two of them in panel b. The start of the next sentence, "Due to all of data is recorded during ..." is unclear. Furthermore, the caption does not adequately justify the presence of panel c. Such a discussion can be found in lines 150-152, but should be mirrored in the caption. Finally, in line 102, should "different reaction states" be "difference reaction times"?

Response:

We corrected the caption to

"**Figure 2 | Photo-thermal reaction kinetics. a** the typical relative displacement distributions of MCBJ conductance measurements with the *in-situ* UV irradiation (30 min). The conductance regions of the top, middle and below section are $10^{-4.5}$ ~10^{-6} G_0 (**vhf**), $10^{-3.0}$ ~ $10^{-4.5}$ G_0 (**dha-*6***), and whole region of $10^{-3.0}$ ~ 10^{-6} G_0, respectively. The Gaussian fitting is used to determine the area ratio of M and T, in which the peak marked as T is ascribed to the tunneling traces and M is for the molecular junction traces. **b** The **dha-*6*** percent versus the reaction time. The upright triangle presents the data points determined from the *in-situ* UV irradiation process, and fitted with the exponential function, $P=P_0+A\times\exp(R_0t)$ (black solid curve), to calculate the time constant decay. The inverted triangle shows the *ex-situ* heating process, and is fitted with the exponential function (black dashed curve). **c** 1-dimension (1D) conductance histogram from different states. Blue is for the initial **dha-*6*** state marked with blue solid box in **b**; red is for the state after *in-situ* UV irradiation 120 min marked as red solid box in **b**; black is from the state after the heating 30 min connected with the state marked as black solid box in **b.** The overlapping of blue and black curve suggests a highly reversible **dha-*6*↔vhf** conversion. Due to the limited time for recording during the *in-situ* UV-MCBJ measurements, the numbers of conductance curves for the statistical analysis are limited to around 500. **d** Plateau distribution of the break junctions from different reaction

times connected to **b**) The conductance region of **vhf** is $10^{-4.5}$ ~10^{-6} G_0 , and the ***dha-6*** is $10^{-3.0}$ ~ $10^{-4.5}$ G_0. ”

11) In lines 108-109, approximately how many traces were neither dha or vhf, or were indeterminate?

Response:

The percentage depends on the progress of the reaction, as shown in Figure 2d. In the initial state, more than 95% of traces (480 in 500 traces) could be assigned to dha junction while 5% of traces (20 in 500 traces) are indeterminate, which are mostly direct tunneling through solvent without formation of molecular junction. At vhf state, the percentage of indeterminate traces varied to ~10%.

To clarify this point, we replace “some traces” by “around 5-10% traces” in the main text.

12) It is stated in line 128 that the irradiation reaction reaches saturation after 80 minutes, but Figure 2b suggests the time is closer to 150 minutes.

Response:

The previous description of 80 minutes is determined from the inflection point of curve. To avoid the confusion and have better description, we change the description to 120 minutes.

13) In line 129, “hollow triangles” should be “upright triangles”, as both symbols are hollow.

Response:

Thanks for your correction. We replaced the “hollow triangles” with “upright triangles”.

14) Is Figure 5 necessary?

Response:

Yes, we believe that it adds experimental evidence to how the **dha** takes a boat conformation (and the position of the anchoring group is therefore very important; strong deviation from co-planarity is seen in the dha-***7*** isomer).

REVIEWERS' COMMENTS:

Reviewer #1 (Remarks to the Author):

The authors have made an extensive revision of the manuscript along the remarks of the Referees, including the addition of new experimental results and calculations, demonstrating that dha-6 is more stable in a junction configuration than dha-7. Still, there are a few formal points that I have to criticize:

(1) It is still disturbing to me, that the authors fit an unphysical, exponentially diverging function to the data on the right side of Fig. 2b. Though a short sentence clarifies this issue, but I am still afraid that some readers would be confused by this fitting curve. As it has important physical meaning that the conventional exponential saturation is far off the data, I would recommend to plot this figure as it was done in the author's response to me, also showing the exponentially saturating (but bad) fitting curve. And a minor remark: the authors should rather define the exponentially saturating fitting function as P_0+A*exp(-R_0*t).

(2) In accordance with my previous report, I still have to express my concern about the correlation plot in Fig. 1d. I am certain, that this is not a 2D correlation histogram, as it was defined by Eq. 4 in Ref. 21. By definition a correlation histogram should have perfect correlation (=1) at the entire diagonal. I do not understand the sentence in the response of the authors "Thus perfect correlation could be demonstrated at the diagonal as shown in the black line with blue color", especially that the black color corresponds to a correlation of -1 according to the colorscale. I suspect that the black line is just a guide to the eye, but the correlation plot does not give perfect correlation at the diagonal, especially in the region -2<log(G/G_0)<0. Probably it is a covariance plot (Eq. 3 in Ref 21), but then the scale is strange, as for a covariance plot there is no reason to scale between -1 and 1. Maybe the authors use some arbitrary scaling. It is OK if the authors use other algorithm than a 2D correlation plot, but then it should be named and defined correctly.

Once the authors correct these issues I can recommend the publication in Nature Communications. Especially my second point is critical: though it is a minor issue compared to the great achievements in the paper, but a publication in Nature Communications should be clear and precise.

Reviewer #2 (Remarks to the Author):

The authors have adequately responded to my earlier comments, as well as those of the other reviewer. This manuscript is much stronger, and I recommend publishing it in Nature Communications. The authors have strengthened and clarified their primary message: Molecular junctions can change chemical reaction pathways. This idea is novel and of great value to the community. I have several minor suggestions that the authors may want to consider before the manuscript is finalized. Further review is not necessary.

1) It may help the reader to refer to Figure 1c (and similar figures elsewhere) as 2D conductance-displacement histograms. Adding the word “displacement” distinguishes this type of analysis from the 2D correlation histograms that are also presented.

2) The figure on page 4 of the response to reviewers (showing the different fits of the kinetic data) should be included in the supporting information. I think having this figure on hand will help support the sentence that was added in lines 151-153 of the main text.

3) Can the authors comment on the dha-7 region of conductance in the correlation analysis? As is, Figure 2d shows that traces can be classified as dha-6 or vhf (or neither) because the two are anti-correlated. Does this same trend hold for dha-7? A brief discussion of this point would make the argument in lines 181-192 (roughly) more compelling.

4) The absolute energies in Supplementary Table 1 are not necessary and, with 9 significant figures, look bizarre. The two columns with relative energies are sufficient.

5) The paper could still use a good proofreading. There were quite a few places where verb tenses didn’t match up, particularly around text added or changed in response to our earlier comments.

Point-by-point response to the referees' comments

REVIEWERS' COMMENTS:

Reviewer #1 (Remarks to the Author):

The authors have made an extensive revision of the manuscript along the remarks of the Referees, including the addition of new experimental results and calculations, demonstrating that dha-6 is more stable in a junction configuration than dha-7. Still, there are a few formal points that I have to criticize:

(1) It is still disturbing to me, that the authors fit an unphysical, exponentially diverging function to the data on the right side of Fig. 2b. Though a short sentence clarifies this issue, but I am still afraid that some readers would be confused by this fitting curve. As it has important physical meaning that the conventional exponential saturation is far off the data, I would recommend to plot this figure as it was done in the author's response to me, also showing the exponentially saturating (but bad) fitting curve. And a minor remark: the authors should rather define the exponentially saturating fitting function as P_0+A*exp(-R_0*t).

Reply:

Thanks for suggestion. We agree to use the function of $P=1-(1-P_0)\times\exp(-R_0t)$ instead of the previous exponential fitting in Figure 3b (after renumbering of Figures), and also modify the caption accordingly.

We also modified the discussion to

"Furthermore, the function of $P=1-(1-P_0)\times\exp(-R_0t)$ was applied for the fitting of the thermal process, and the disagreement between the fitting and the experiments suggests that the constraints of the junction may alter the kinetics of the back reaction."

We also modified the exponentially saturating fitting function as suggested by the reviewer.

(2) In accordance with my previous report, I still have to express my concern about the correlation plot in Fig. 1d. I am certain, that this is not a 2D correlation histogram, as it was defined by Eq. 4 in Ref. 21. By definition a correlation histogram should have perfect correlation (=1) at the entire diagonal. I do not understand the sentence in the response of the authors "Thus perfect correlation could be demonstrated at the diagonal as shown in the black line with blue color", especially that the black color corresponds to a correlation of -1 according to the colorscale. I suspect that the black line is just a guide to the eye, but the correlation plot does not give perfect correlation at the diagonal, especially in the region -2<log(G/G_0)<0. Probably it is a covariance plot (Eq. 3 in Ref 21), but then the scale is strange, as for a covariance plot there is no reason to scale between -1 and 1. Maybe the authors use some arbitrary scaling. It is OK if the authors use other algorithm than a 2D correlation plot, but then it should be named and defined correctly.

Reply:

We highly appreciate for the careful corrections from the reviewer. Yes, we check our analysis code that the histogram is actually not 2D correlation histogram but scaled covariance histogram as suggested by the reviewer. We apply the algorithm $H_{i,j}^{cov} =$

$\langle \delta N_i(r) \cdot \delta N_j(r) \rangle_r$ (Eq. 3 in Ref 21) to construct the covariance matrix and then the average of the covariance in the molecular junction regime (from 10^{-3} G_0 to 10^{-6} G_0, and the average value is determined to be around 3.2 for this set of data) are applied for scaling to have better visualization. To avoid the confusion, we remove the scaling factor to redraw the figure 2b, and we also removed the black line originally as guideline for eyes to avoid the confusion with the negative covariance.

We have also tried to reanalyze the data with 2D correlation histogram, however, it seems the 2D correlation could not provide further information beyond the current 2D covariance histogram that we prefer to use the 2D covariance histogram to present in this paper.

We updated the figure and figure captions, and also corrected the term from 2D correlation to 2D covariance histogram in the main text.

Once the authors correct these issues I can recommend the publication in Nature Communications. Especially my second point is critical: though it is a minor issue compared to the great achievements in the paper, but a publication in Nature Communications should be clear and precise.

Reviewer #2 (Remarks to the Author):

The authors have adequately responded to my earlier comments, as well as those of the other reviewer. This manuscript is much stronger, and I recommend publishing it in Nature Communications. The authors have strengthened and clarified their primary message: Molecular junctions can change chemical reaction pathways. This idea is novel and of great value to the community. I have several minor suggestions that the authors may want to consider before the manuscript is finalized. Further review is not necessary.

1) It may help the reader to refer to Figure 1c (and similar figures elsewhere) as 2D conductance-displacement histograms. Adding the word "displacement" distinguishes this type of analysis from the 2D correlation histograms that are also presented.

Reply:

We agree and now we modify the caption to be "2D conductance-displacement histograms".

2) The figure on page 4 of the response to reviewers (showing the different fits of the kinetic data) should be included in the supporting information. I think having this figure on hand will help support the sentence that was added in lines 151-153 of the main text.

Reply:

Thanks for suggestions, the new fitting are now employed in Figure 2b as also suggested by the first reviewer.

3) Can the authors comment on the dha-7 region of conductance in the correlation analysis? As is, Figure 2d shows that traces can be classified as dha-6 or vhf (or neither) because the two are anti-correlated. Does this same trend hold for dha-7? A brief discussion of this point would make the argument in lines 181-192 (roughly) more compelling.
Reply:

Thanks for suggestions. We checked the covariance analysis of dha-7 data that there is also anti-correlation observed similar to dha-6 but unfortunately the 2D covariance analysis is not as significant as for dha-6, which is mainly due to the large conductance variation of dha-7. Thus we prefer not to present the data.

4) The absolute energies in Supplementary Table 1 are not necessary and, with 9 significant figures, look bizarre. The two columns with relative energies are sufficient.

Reply:

We agree to remove the absolute energies column in the table.

5) The paper could still use a good proofreading. There were quite a few places where verb tenses didn't match up, particularly around text added or changed in response to our earlier comments.

Reply:

Thanks for suggestions, now we have again proofreading to avoid mismatches.
